# Seasonal dynamic modeling for real-time prediction of human brucellosis epidemiological trends in Gansu, Guangdong and Sichuan Provinces, China

**DOI:** 10.1371/journal.pntd.0014443

**Published:** 2026-06-25

**Authors:** Pengwei Lou, Yuting Huang, Jiandong Yang, Nannan Chen, Feng Zhao, Jiabo Xu, Kai Wang

**Affiliations:** 1 College of Information Engineering, Xinjiang Institute of Engineering, Urumqi, China; 2 Department of Medical Administration, Traditional Chinese Medicine Hospital Affiliated With Xinjiang Medical University, Urumqi, China; 3 Department of Infectious Disease Control and Prevention, Urumqi Center for Disease Control and Prevention, Urumqi, China; 4 School of Public Health, Xinjiang Medical University, Urumqi, China; 5 College of Medical Engineering and Technology, Xinjiang Medical University, Urumqi, China; University of Dhaka, BANGLADESH

## Abstract

**Objectives:**

Construct a seasonal dynamic model that captures the transmission characteristics of brucellosis in Gansu, Guangdong and Sichuan provinces of China, in order to fit and predict the epidemic trends of new human brucellosis, and further formulate scientific and targeted prevention and control strategies.

**Methods:**

Based on the number of new human brucellosis cases reported by the Centers for Disease Control and Prevention in Gansu, Guangdong and Sichuan provinces from 2021 to 2024, a seasonal SEIV dynamic model of brucellosis transmission between sheep/cattle and humans was constructed, with parameters estimated by using the nonlinear least squares method and the Markov Chain Monte Carlo method. The model fitted the epidemic trends of new human brucellosis and estimated the basic reproduction number *R*_0_*.* Through parameter sensitivity analysis, effective prevention and control measures can be proposed.

**Results:**

The established seasonal SEIV dynamic model can well fit the number of new human brucellosis cases and cumulative new human brucellosis cases in Gansu, Guangdong and Sichuan provinces, respectively. The calculated mean absolute percentage error (*MAPE*) values were approximately 20% and 6%, respectively, indicating a good agreement between the fitted and actual values. It is projected that Gansu and Sichuan provinces will reach peak values of 146,310 and 157,903 cases in July and May 2038, respectively, followed by a gradual declines toward a stable state. The epidemic in Guangdong province will reach a relatively stable, sustained prevalence by 2038. The estimated basic reproduction number *R*_0_ for brucellosis transmission in Gansu, Guangdong and Sichuan provinces were 2.2510 (95%CI: 2.2160 - 2.2859), 2.7937 (95%CI: 2.7592 - 2.8283) and 2.9499 (95%CI: 2.9007 - 2.9992), respectively. These findings indicate that brucellosis will continue to spread under the current prevention and control measures. Finally, sensitivity analyses of the number of new human brucellosis cases and *R*_0_ were conducted based on specific parameters, it is demonstrated that increasing the culling rate of infected sheep/cattle, raising the vaccination rate of susceptible sheep/cattle, and reducing the immune loss rate of vaccinated sheep/cattle can effectively suppress the spread of brucellosis.

**Conclusion:**

The constructed seasonal SEIV dynamic model can accurately simulates the epidemic trends of new human brucellosis cases in Gansu, Guangdong and Sichuan provinces, quantitatively proposes effective prevention and control measures, and lay a theoretical foundation for combating the spread of brucellosis.

## Author summary

Brucellosis is a zoonotic disease that seriously endangers the health of both humans and livestock. The disease remains uncontrolled in China, showing an upward trend year by year, new human brucellosis cases are reported almost every month, with sudden surges occurring particularly in southern cities. To accurately assess the transmission intensity of brucellosis, simulate and predict fluctuations in human brucellosis cases incidence, and propose quantitative and effective control measures, we conducted a comprehensive analysis of the transmission mechanism of brucellosis between sheep/cattle and humans. Based on the seasonal variation characteristics of the latest monthly new human brucellosis cases in Gansu, Guangdong and Sichuan provinces in China, we constructed a seasonal SEIV dynamic model with periodic transmission rates. The model can accurately simulate the number of new human brucellosis cases in Gansu, Guangdong, and Sichuan provinces, enabling both short-term validation and long-term prediction. This confirms that the model developed in this study is applicable to the transmission of brucellosis across Chinese provinces. Through sensitivity analysis of model parameters, we have summarized effective measures to control the spread of brucellosis: increasing the culling rate of infected sheep/cattle and the vaccination rate of susceptible sheep/cattle, while simultaneously reducing the immune loss rate of vaccinated sheep/cattle. Our research findings provide a scientific theoretical basis for controlling the spread of brucellosis across Chinese provinces.

## 1. Introduction

Brucellosis is a prevalent zoonotic infectious disease caused by brucella, affecting both humans and animals [1]. Brucella bacteria encompass a wide range of species, including B. melitensis, B. abortus, B. suis, B. canis and B. ovis. Infected sheep and cattle are the primary sources of transmission [2]. The World Organization for Animal Health (OIE) classifies brucellosis as a Category B animal infectious disease, and the Law of the People’s Republic of China on the Prevention and Treatment of Infectious Diseases also designates it as a Category B infectious disease [3]. Globally, approximately 500,000 new human brucellosis cases are reported annually, among which China accounting for around 60,000 cases, representing roughly 12% of the global total [4].

Brucellosis is mainly transmitted among livestock and from infected animals to humans, while human-to-human transmission is extremely rare [5,6]. People primarily contract the disease through contact with infected livestock and their products. The main clinical symptoms of human brucellosis include fever, excessive sweating, fatigue, joint and muscle pain, and in some cases, hepatosplenomegaly, testicular enlargement, etc, severe cases may lead to loss of working capacity [7]. Most patients have a generally favorable prognosis with accurate diagnosis and timely treatment. Without prompt intervention, the condition may progress from acute to chronic stages. Chronic cases become difficult to cure, severely impair work capacity. Brucellosis itself rarely causes death [8,9].

The first human case of brucellosis in mainland China were reported in 1905. The incidence rate remained quite severe until 1980, declined during 1980–1990, but rebounded after 1995. Currently, the transmission situation of brucellosis remains severe [10]. Since 1950, comprehensive prevention and control measures against brucellosis have been gradually implemented in mainland China; nevertheless, the overall control effect remains unsatisfactory [11]. Beyond temporal trends, the prevalence of human brucellosis in mainland China also exhibits distinct spatial characteristics. Affected regions have gradually expanded from northern pastoral areas to southern China, with several southern provinces having become endemic since 2004 [12,13]. In recent years, China’s livestock population has increased sharply to meet the growing domestic meat demand. Extensive cross-provincial livestock transportation, particularly from northern pastoral regions to southern provinces, has heightened the mobility of infected animals. Brucellosis keeps spreading among livestock, and direct or indirect contact with infected animals remains the primary cause of human brucellosis epidemics in southern provinces [14]. In the study by Jiang et al. [15], the results revealed that northern and eastern China (Inner Mongolia and Shanxi) shared the same MLVA-16 genotype as southern regions (Guangdong). This also confirms that part of brucellosis in southern regions is caused by the introduction of infected animals from other regions. Thus, controlling the prevalence of brucellosis in animal hosts is crucial for controlling human brucellosis.

Mathematical models have become valuable tools for further exploring the transmission dynamics of infectious diseases, with particular application to brucellosis. Models constructed according to the transmission mechanisms of brucellosis can be used to predict epidemic trends and transmission patterns, assess transmission intensity, optimize prevention and control strategies, evaluate the efficacy of intervention measures, and provide a scientific basis for precise prevention and control of the disease [16]. In recent years, most existing studies have mainly focused on brucellosis transmission dynamic models within livestock populations. Only a few scholars have constructed cross-species transmission models from livestock to humans, analyzed the impacts of diverse control measures on human brucellosis prevalence, and obtained relevant quantitative and qualitative results. Zinsstag et al. [17] analyzed the epidemiological status of brucellosis in Mongolia, established a coupled sheep-cattle-human three-population dynamic model, predicted the epidemic trend of brucellosis, and confirmed that vaccinating sheep/cattle is an effective strategy for controlling the transmission of brucellosis. Zhang et al. [18] considered the transmission mechanism of brucellosis between sheep and humans in Jilin province and established a dynamic model to evaluate the effectiveness of vaccination, detection-culling, and tracking-culling of sheep. Hou et al. [19] proposed a dynamic model for the sheep-human transmission of brucellosis in Inner Mongolia, and found that vaccination and eliminating strategies targeting adult sheep are effective measures for controlling brucellosis. Ma et al. [20] leverage a discrete-time human-sheep coupled model constructed by employing backward Euler method to investigate the effects of different control measures on the brucellosis transmission in Jilin province. Lastly, they revealed that increasing the frequency and effectiveness of disinfection, strengthening the efficacy of health education and publicity, and raising the elimination rate can all shorten the epidemic duration of brucellosis and reduce the final outbreak scale. Although such models can reasonably predict the prevalence trends of human brucellosis and provide corresponding prevention and control recommendations, they fail to incorporate the seasonal patterns of the disease. All simulations and projections were based on annual data, which cannot adequately reflect the seasonal transmission characteristics and epidemic patterns of brucellosis. Furthermore, the lag in official released data undermines the efficacy of prevention and control measures. Accordingly, this study draws upon the model proposed in Reference [19], and incorporates the seasonal incidence pattern of brucellosis to explore its transmission dynamics in Gansu, Guangdong, and Sichuan provinces of China. In dynamic models, when transmission rates exhibit seasonal fluctuations, sine functions can effectively fit the periodic trends of epidemiological data by adjusting amplitude and phase parameters. This approach has been widely applied to dynamics models of seasonal infectious disease, including schistosomiasis, hand-foot-and-mouth disease (HFMD), rabies, and tuberculosis, with satisfactory predictive performance achieved [21–24]. We selected Gansu, Guangdong, and Sichuan provinces as our primary research areas. The provincial Center for Disease Control and Prevention in these regions releases monthly surveillance data on new human brucellosis cases timely, which guarantees the reliability of the established model and the rationality of the proposed control strategies. Additionally, Guangdong and Sichuan provinces are located in southern China, with fewer cattle and sheep raised compared with Gansu province in the northwest China, making it possible to verify the general applicability of the constructed model.

The objective of this study is to construct a seasonal dynamic model with periodic transmission rates for sheep/cattle-human coupling, based on the latest monthly data on new human brucellosis cases published in Gansu, Guangdong and Sichuan provinces, and incorporating the transmission mechanisms of brucellosis. Subsequently, the model is used to predict human brucellosis epidemic trends and quantify the effectiveness of prevention and control measures, thereby providing a theoretical basis for controlling brucellosis in China. The feasibility of this study is summarized as follows: (1) No studies have reported the use of dynamic model to analyze the transmission characteristics and patterns of brucellosis in Gansu, Guangdong and Sichuan provinces. (2) Although the number of new human brucellosis cases reported in Guangdong and Sichuan provinces remains relatively low, it has shown a increasing trend year by year. Therefore, proposing effective control measures to curb brucellosis transmission is of great significance for preventing disease outbreaks. (3) Brucellosis transmission exhibits obvious seasonal characteristics. Incorporating transmission rates parameters into the model can more accurately reflect the transmission patterns and epidemic trends of brucellosis.

The paper is organized as follows. The research background of brucellosis and the application of dynamic modeling methods are presented in Section 1. Section 2 determines the number of new human brucellosis cases and seasonal analysis in Gansu, Guangdong and Sichuan provinces from 2021 to 2024. In Section 3, we first construct a seasonal SEIV dynamic model of brucellosis transmission between sheep/cattle and humans, and determine the parameter values. Then, the model simulates and predicts the epidemiological trends of new human brucellosis cases in Gansu, Guangdong and Sichuan provinces, respectively. Finally, we estimate the basic reproduction number *R*_0_ for brucellosis transmission and propose effective control measures through parameter sensitivity analysis. Section 4 discusses and summarizes the article.

## 2. Materials and methods

### 2.1. Data sources

**Fig 1:**
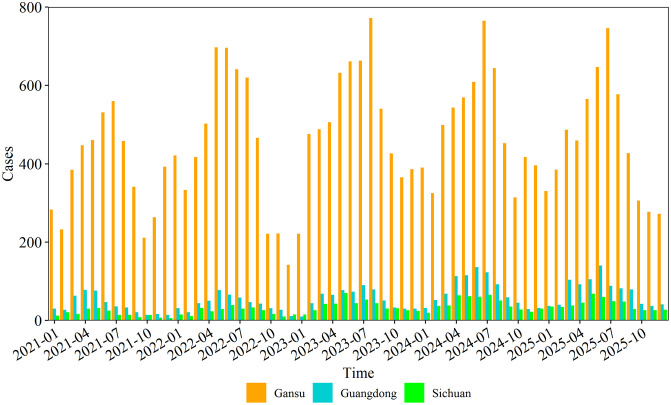
The number of new human brucellosis cases in Gansu, Guangdong and Sichuan provinces from January 2021 to December 2025.

Monthly data on new human brucellosis cases from January 2021 to December 2025, as published by the Centers for Disease Control and Prevention (CDC) of Gansu, Guangdong, and Sichuan provinces, are presented in Fig 1 [25–27]. These three provinces in China are notable for their timely reporting of infectious disease data, with monthly updates on new human brucellosis cases. The cumulative reported cases during the study period were 27,477 in Gansu, 3,394 in Guangdong, and 1,922 in Sichuan, all of which exhibited a year-on-year increasing trend. In this study, a seasonal dynamic model was established using the monthly number of new human brucellosis cases between 2021 and 2024, and the 2025 dataset was subsequently employed to validate the model’s predictive performance.

### 2.2. Seasonal analysis

**Fig 2:**
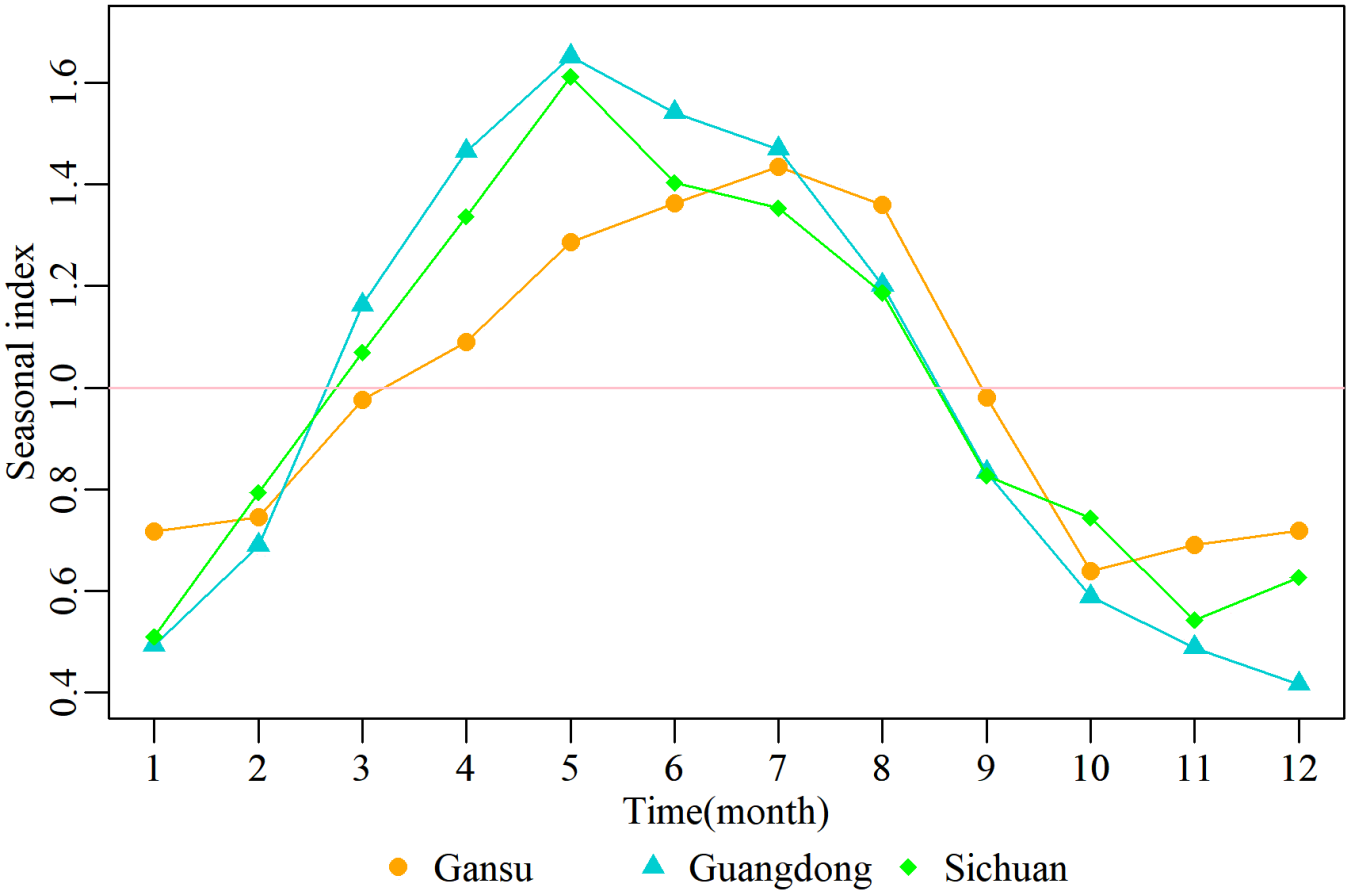
Seasonal index of new human brucellosis in Gansu, Guangdong and Sichuan provinces.

The seasonal index is used to verify whether an infectious disease has seasonal incidence, with the calculation formula $S_{j}={\overset{―}{x}}_{j}/\overset{―}{x}$, where $\overset{―}{x}$ represents the total average incidence of the infectious disease, and ${\overset{―}{x}}_{j}$ represents the average incidence of the infectious disease in the *j*-th month [28]. The seasonal indexes of new human brucellosis cases in Gansu, Guangdong and Sichuan provinces are shown in Fig 2. From March to August, $S_{j}$ > 1, indicating that the incidence of human brucellosis is higher in spring and summer, with obvious seasonality. Guangdong and Sichuan provinces have the highest incidence in May each year, while Gansu province primarily experiences its peak in July.

## 3. Results

### 3.1. Model formulation and derivation of the basic reproduction number *R*_0_

**Fig 3:**
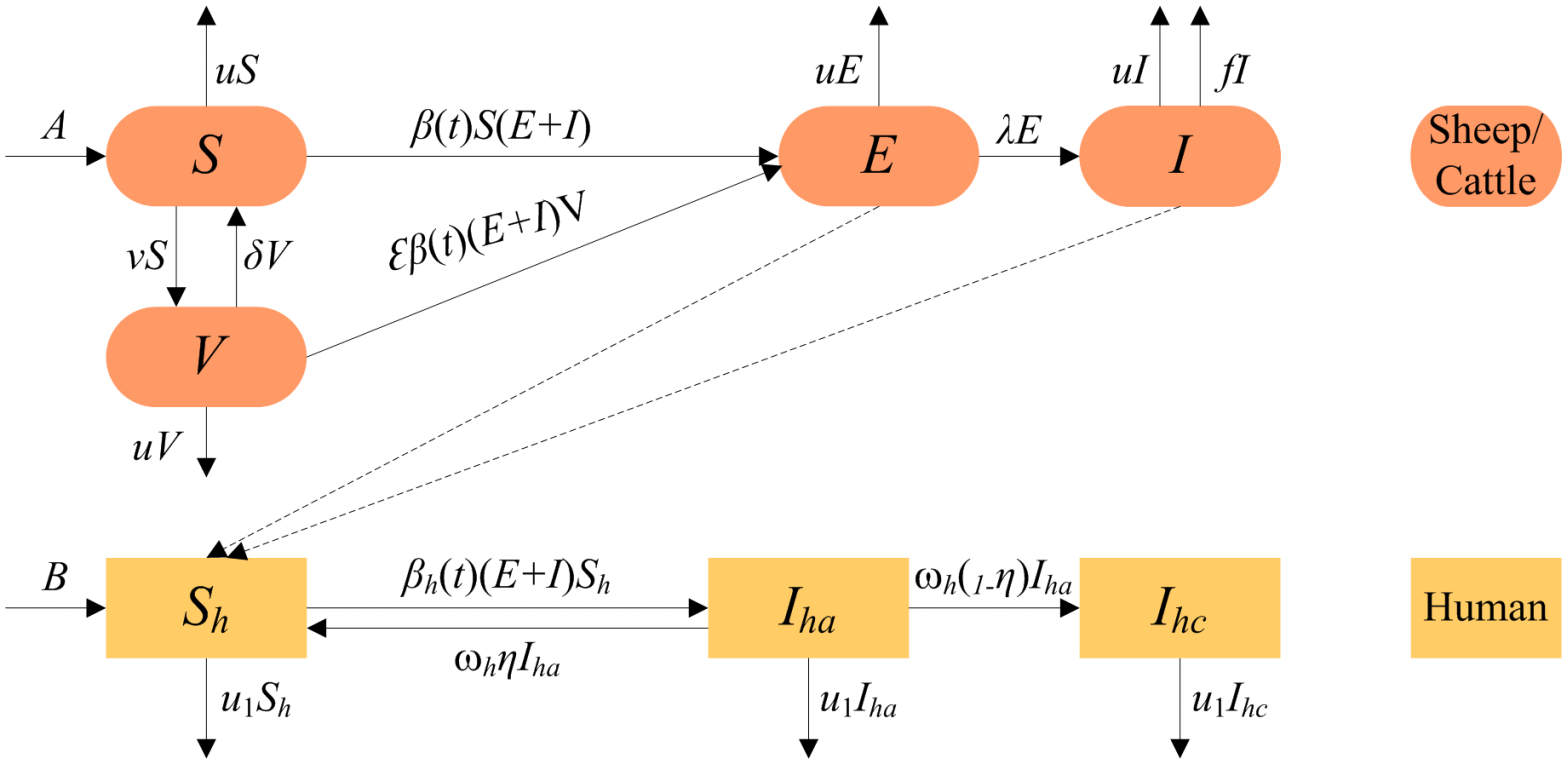
The transmission diagram of brucellosis.

A dynamic model for brucellosis transmission in sheep/cattle and from sheep/cattle to humans was developed based on the transmission mechanism of brucellosis and Reference [19]. Specifically, sheep/cattle are divided into four subgroups: susceptible *S*(*t*), exposed *E*(*t*), infected *I*(*t*) and vaccinated *V*(*t*). Humans are divided into three subgroups: susceptible *S*_*h*_(*t*), acute infected *I*_*ha*_(*t*) and chronic infected *I*_*hc*_(*t*). The assumptions of the brucellosis transmission model are as follows: (1) The incubation period of human brucellosis is approximately 2 weeks. In the early stage of infection, the primary clinical symptom in humans is fever, and many patients are misdiagnosed, leading to ineffective treatment and progression to the acute infection stage at the time of diagnosis. Therefore, this study assumes that upon infection, humans directly progress to the acute infected state. (2) As there are few reports of brucellosis transmission between humans, the model assumes no human-to-human transmission. (3) Human brucellosis is predominantly transmitted via direct contact with infected sheep/cattle, while the transmission contribution of brucella in the natural environment remains negligible. Therefore, environmental factors are not considered in this model. (4) In sheep/cattle, antibody levels gradually decline over time following vaccination. Moreover, the protective efficacy of the vaccine is limited, and the animals may fail to mount a sufficiently robust immune response, thereby remaining at risk of infection. Consequently, vaccinated sheep/cattle may still become susceptible or exposed individuals. The flowchart of brucellosis transmission is shown in Fig 3.

Based on the flow chart of brucellosis transmission, the following ordinary differential Equation (1) can be established. In model (1), only the first four equations for the transmission of brucellosis by sheep/cattle need to be considered.


$$\begin{array}{c} \left\{ \begin{array}{c} @l@\frac{dS}{dt}=A-\beta \left(t\right)S\text{(}E+I\text{)}-\text{(}\mu +\nu \text{)}S+\delta V\\ \frac{dE}{dt}=\beta \left(t\right)\text{(}S+\varepsilon V\text{)(}E+I\text{)}-\text{(}\lambda +\mu \text{)}E\\ \frac{dI}{dt}=\lambda E-\text{(}\mu +f\text{)}I\\ \frac{dV}{dt}=\nu S-\text{(}\mu +\delta \text{)}V-\varepsilon \beta \left(t\right)V\text{(}E+I\text{)}\\ \frac{dS_{h}}{dt}=B-\beta_{h}\left(t\right)S_{h}(E+I)+\omega_{h}\eta I_{ha}-\mu_{\text{1}}S_{h}\\ \frac{dI_{ha}}{dt}=\beta_{h}\left(t\right)S_{h}\text{(}E+I\text{)}-\omega_{h}I_{ha}-\mu_{\text{1}}I_{ha}\\ \frac{dI_{hc}}{dt}=\omega_{h}\text{(}1-\eta \text{)}I_{ha}-\mu_{\text{1}}I_{hc} \end{array}\right. \end{array}$$ (1)

It is clear that the first four equations of model (1) has a unique positive disease-free equilibrium $P_{\text{0}}=(S^{*},\;\text{0},\;\text{0},\;V^{*})$, where


$$\begin{array}{c} S^{*}=\frac{A(\mu +\delta )}{\mu (\mu +\nu +\delta )},\;V^{*}=\frac{A\nu }{\mu (\mu +\nu +\delta )}. \end{array}$$ (2)

Consider the following auxiliary equations:


$$\begin{array}{c} \left\{ \begin{array}{c} @l@\frac{dx}{dt}=\text{A}-(\mu +\nu )x+\delta y,\;\\ \frac{dy}{dt}=\nu x-(\mu +\delta )y. \end{array}\right.\; \end{array}$$ (3)

In fact, the Jacobian matrix of model (3) at equilibrium (*A*(*μ* + *δ*)/*μ*(*μ* + *v* + *δ*), *Av/μ*(*μ* + *v* + *δ*)) is


$$\begin{array}{c} J=\left(\begin{array}{c} @l@-(\mu +\nu )\;\delta \\ \;\nu \;-(\mu +\delta ) \end{array}\right)\; \end{array}$$ (4)

and then the corresponding characteristic equation is


$$\begin{array}{c} \Phi \left(\lambda \right)=\lambda^{\text{2}}+(2\mu +\delta +\nu )\lambda +(\mu +\delta +\nu )\mu . \end{array}$$ (5)

By simple calculation, it is easy to obtain the two roots of Φ(λ) are $\lambda_{\text{1}}=-\mu \;$and $\lambda_{\text{2}}=-(\mu +\nu +\delta )$. Hence, we obtain the unique positive equilibrium (*A*(*μ* + *δ*)/*μ*(*μ* + *v* + *δ*), *Av/μ*(*μ* + *v* + *δ*)) is locally asymptotically stable. In addition, since model (3) is linear, by the theorems of stability of the differential equations, we obtain the equilibrium (*A*(*μ* + *δ*)/*μ*(*μ* + *v* + *δ*), *Av/μ*(*μ* + *v* + *δ*)) is globally asymptotically stable, which completes the proof.

Now, we employ the method outlined by Wang et al. [29,30] to calculate the basic reproduction number *R*_0_ for the first four equations of model (1). Let


$$\begin{array}{c} f\left(t,\;x\right)=\left(\begin{array}{c} @l@\beta \left(t\right)(S+\varepsilon V)(E+I)\\ \;0\\ \;0\\ \;0 \end{array}\right),\;v\left(t,\;x\right)=\left(\begin{array}{c} @l@(\lambda +\mu \text{)}E\\ \text{(}\mu +f\text{)}I-\lambda E\\ \beta \text{(}t\text{)}S\text{(}E+I\text{)}+\text{(}\mu +\nu \text{)}S-A-\delta V\\ \text{(}\mu +\delta \text{)}V+\varepsilon \beta \text{(}t\text{)}V\text{(}E+I\text{)}-\nu S \end{array}\right),\; \end{array}$$ (6)

where $\text{x}=(E,I,S,V{)}^{T}$, and then the first four equations of model (1) takes the following form


$$\begin{array}{c} \dot{x}\left(t\right)=f\left(t,\;x\right)-v\left(t,\;x\right)\;\underset{―}{\underset{―}{\Delta }}\;f\left(t,\;x\left(t\right)\right). \end{array}$$ (7)

Obviously, model (7) has a disease-free equilibrium x^*^(*t*) = (0, 0, S^*^, V^*^).

Next, we set two 2 × 2 matrices as follows:


$$\begin{array}{c} F\left(t\right)={\left(\frac{\partial f_{i}\left(t,\;x^{*}\left(t\right)\right)}{\partial x_{j}}\right)}_{1\leq i,j\leq 2},\;V\text{(}t\text{)}={\left(\frac{\partial v_{i}\left(t,\;x^{*}\left(t\right)\right)}{\partial x_{j}}\right)}_{1\leq i,j\leq 2}, \end{array}$$ (8)

Where $f_{i}\left(t,\;x\left(t\right)\right)$ and $v_{i}\left(t,\;x\left(t\right)\right)$ are the *i*th component of $f_{i}\left(t,\;x\left(t\right)\right)$and $v_{i}\left(t,\;x\left(t\right)\right)$, respectively. Then, by simple computation, it follows that


$$\begin{array}{c} F\left(t\right)=\left(\begin{array}{c} @l@\beta \left(t\right)(S^{*}+\varepsilon V^{*})\;\beta \text{(}t\text{)(}S^{*}+\varepsilon V^{*}\text{)}\\ \text{0 0} \end{array}\right),\;V\left(t\right)=\left(\begin{array}{c} @l@\lambda +\mu \;0\\ -\lambda \;\mu +f \end{array}\right).\; \end{array}$$ (9)

Hence, we easily check that conditions (A1) - (A7) given in [29] are satisfied.

Let *Y*(*t*, *s*) be the 2 × 2 matrix solution of the following initial value problem:


$$\begin{array}{c} \frac{d}{dt}Y\left(t,\;s\right)=-V\left(t\right)Y\left(t,\;s\right)\;\forall t\geq s,\;Y\left(s,s\right)=I. \end{array}$$ (10)

Let $C_{\omega }$ be the ordered Banach space of all ω-periodic continuous function form *R* to *R*^2^ with the maximum norm ‖·‖. The positive cone $\overset{+}{\underset{\omega }{C}}=\left\{\varphi \in C_{\omega }:\varphi \left(t\right)\geq 0for\;allt\in R\right\}$. Suppose $\varphi \left(s\right)\in C_{\omega }$ is the initial distribution of infectious individuals in this periodic environment, then F(s)φ(s) is the rate of new infectious individuals produced by the infected individuals who were introduced at time *s*, and Y(t,s)F(s)φ(s) represents the distributions of those infected individuals who were newly infected at time *s* and remain in the infected compartment at time *t* for *t* ≥ *s*. Hence, we define a linear operator $L:C_{\omega }\to C_{\omega }$as follows:


$$\begin{array}{c} (L\varphi )(t)=\int_{\text{0}}^{+\infty }Y\text{(}t\text{,}t-a\text{)}F\text{(}t-a\text{)}\varphi \text{(}t-a\text{)}da\forall t\in R\text{,}\varphi \in C_{\omega }. \end{array}$$ (11)

The operator *L* is positive, continuous, and compact on $C_{\omega }$. Thus, *R*_0_ can be characterized by the existence of a nonnegative and nonzero $\varphi \in \overset{+}{\underset{\omega }{C}}$ such that


$$\begin{array}{c} \left(L\varphi \right)\left(t\right)=R_{\text{0}}\varphi \left(t\right). \end{array}$$ (12)

Now, we define basic reproduction number *R*_0_ for model (7) by


$$\begin{array}{c} R_{\text{0}}=\rho \left(L\right), \end{array}$$ (13)

where ρ(L) is the spectral radius of *L*.

### 3.2. Model parameter values and descriptions

In the equations of model (1) for brucellosis transmission in sheep/cattle, *A* represents the monthly new recruitment of sheep/cattle; *μ* represents the natural mortality rate of sheep/cattle; *ν* represents the vaccination rate of susceptible sheep/cattle; *δ* represents the immune loss rate of vaccinated sheep/cattle; *ε* represents the ineffective vaccination rate; *λ* represents the transition rate from exposed to infected sheep/cattle; *f* represents the culling rate of infected sheep/cattle. In the process of human brucellosis infection, *B* represents the monthly new recruitment of human; η represents the cure rate from acute infected individuals to susceptible individuals; $\omega_{h}$ represents the removal rate from acute infected individuals; $\mu_{\text{1}}$ represents the natural mortality rate of humans. The value descriptions of each parameter are shown in Table 1. In model (1), the periodic transmission rate of brucellosis from susceptible sheep/cattle *S*(*t*) to exposed individuals *E*(*t*) is β(t)=α[1+bsin(ωt+c)], and the periodic transmission rate of brucellosis from exposed and infected sheep/cattle to humans is $\beta_{h}\left(t\right)=\alpha_{h}\left[1+b_{h}\;sin\left(\omega t+c_{h}\right)\right]$. Both β(t) and $\beta_{\text{h}}\left(t\right)$ can be represented by sine func*t*ions [31]. To reflect *t*he annual seasonal characteristics of brucellosis transmission, we set the transmission cycle as one year. Mathematically, the period *T* of a sinusoidal function is related to its angular frequency *ω* by *T* = 2*π*/*ω*. With monthly time units, setting *ω* = *π*/6 yields a period *T* = 12, corresponding exactly to 12 months. This parameter ensures that the transmission rates β(t) and $\beta_{h}\left(t\right)$ complete one full annual cycle. Parameters *a* and *a*_*h*_ represent baseline contact rates, *b* and *b*_*h*_ represent amplitudes, *c* and *c*_*h*_ represent phases. Specific parameter values can be reasonably estimated by fitting actual incidence data using Bayesian Markov Chain Monte Carlo (MCMC) methods.

**Table 1:** Definitions and values of model parameters.

Parameter	Unit	Value	Comments	Reference
*A*	month^-1^	Table 3	Monthly new recruitment of sheep/cattle	[A]
μ	month^-1^	0.0185	The natural mortality rate of sheep/cattle	[B]
*ƒ*	month^-1^	0.0163 (Range: 0.0125 - 0.0313)	The culling rate of infected sheep/cattle	[C]
*δ*	month^-1^	0.0333	The immune loss rate of vaccinated sheep/cattle	[33]
*ν*	month^-1^	0.0236 (Range: 0.0209 - 0.0263)	The vaccination rate of susceptible sheep/cattle	[C]
*ε*	month^-1^	0.0150	The ineffective vaccination rate	[19]
*λ*	month^-1^	2	The transition rate from exposed to infected sheep/cattle	[28]
*a*	–	Table 3	Baseline contact rate	Fitting
*b*	–	Table 3	Amplitudes	Fitting
*c*	–	Table 3	Phases	Fitting
*B*	month^-1^	Table 3	Monthly new recruitment of human	[D]
$\mu_{\text{1}}$	month^-1^	Table 3	The natural mortality rate of human	[D]
*η*	month^-1^	0.0500	The cure rate from acute infected individuals to susceptible individuals	[34]
$\omega_{h}$	month^-1^	0.1667	The removal rate from acute infected individuals	[19]
*a*_*h*_	–	Table 3	Baseline contact rate	Fitting
*b*_*h*_	–	Table 3	Amplitudes	Fitting
*c*_*h*_	–	Table 3	Phases	Fitting

[A] The China Statistical Yearbook records the sheep/cattle inventory N for Gansu, Guangdong, and Sichuan provinces from 2021 to 2024 [35]. Using the sheep/cattle growth equation \stackrelgN=A−μN, the monthly new recruitment of sheep/cattle *A* was obtained via nonlinear least squares (NLS) estimation, the results are shown in Table 3.

[B] Adult sheep/cattle life span is about 4–5 years, assuming that the individual natural mortality rate *μ* = 1/(4.5 × 12) = 0.0185.

[C] We have determined the *ƒ* values for Inner Mongolia, Xinjiang, Shanxi, Hebei, and Jilin provinces (areas with high incidence of brucellosis), as well as the *ν* values for Inner Mongolia and Xinjiang [19,28,34,36]. Therefore, this study assumes that the monthly values of *f* and *ν* for Gansu, Guangdong, and Sichuan provinces are approximately equal to the average values for other regions, where *f* = (0.0313 + 0.0125 × 4)/5 = 0.0163, with a range of 0.0125 to 0.0313; *ν* = (0.0263 + 0.0209)/2 = 0.0236, with a range of 0.0209 to 0.0263. The values of both parameters fell within the ranges reported in previous studies. Notably, parameter *f* approached its minimum value, ensuring that the proposed control measures could reasonably cover all provinces. Detailed parameter value analysis results are provided in Section 3.7.

[D] The China Statistical Yearbook records the total population *N*_1_ and natural mortality rate $\mu_{\text{1}}$ in Gansu, Guangdong and Sichuan provinces from 2021 to 2024 [35]. Using NLS method based on population growth equation $\mathrm{\backslash stackrel}\text{g}N_{\text{1}}=B-\mu N_{\text{1}}$, the monthly new recruitment of human *B* was estimated, as shown in Table 3.

The number of newly reported human brucellosis cases is modeled as Poisson-distributed random variable, which is suitable for counting events within a given time span. We calibrate the model by sampling from the posterior distribution of parameter vector $\theta |z=\left\{\alpha ,\;b\text{,}c\text{,}\alpha_{h}\text{,}b_{h}\text{,}c_{h},\;E\left(0\right),\;I\left(0\right),\;V\left(0\right)\right\}|z$, where vector *z* represents the number of new human brucellosis cases, and *Z*(*t*) = *Y*(*t*) - *Y*(*t*-1), $\frac{d}{dt}Y\text{(}t\text{)}=\alpha_{h}\left[1+b_{h}\;sin\left(\frac{\pi }{\text{6}}t+c_{h}\right)\right]S_{h}\text{(}E+I\text{)}$. Sampling is implemented via MCMC under *t*he Me*t*ropolis-Has*t*ings acceptance rule. The posterior density is $f_{\Theta \text{|}z}\left(\theta \right|z)=\underset{T}{\Pi }L(Y\left(t\right)\left|\theta \right)f_{\Theta }\left(\theta \right)$. The prior density $f_{\Theta }\left(\theta \right)$ is the joint probability of nine univariate priors. The model parameters *a*, *b*, *c*, *a*_*h*_, *b*_*h*_, *c*_*h*_, *E*(0), *I*(0), and *V*(0) follow uniform distributions with ranges as shown in Table 2. Code implementation for model construction and parameter estimation was performed using MATLAB R2024a. We conducted 40,000 MCMC sampling iterations, discarding the first 10,000 samples as the burn-in period. Based on the remaining 30,000 samples, point estimates and 95% confidence intervals for the parameters were calculated. Three parallel MCMC chains were initialized from overdispersed starting values. Chain 1 was set to the currently used optimal parameter estimates derived from the model. The initial values for Chain 2 and Chain 3 were independently drawn from the uniform prior distributions of the parameters. Convergence was assessed using the Gelman-Rubin statistic, with a threshold of \stackrel∧R<1.05. In this study, all parameters yielded \stackrel∧R values below 1.05, indicating successful convergence of all chains, the results are summarized in S1 Table. Visual inspection of trace plots across chains further confirmed satisfactory mixing and stationarity (S1 Fig). The acceptance rates for Gansu, Guangdong, and Sichuan provinces were approximately 68.53%, 66.55%, and 54.45%, respectively, detailed results for each chain are provided in S1 Table. The posterior distribution results for the six parameters in β(t) and $\beta_{\text{h}}\left(t\right)$ are presented in Table 3, and histograms for the individual parameters are also provided (S2 Fig). Autocorrelation decayed gradually to near zero within 45 lags (S3 Fig). The minimum effective sample sizes (ESS) for all parameters in Gansu, Guangdong, and Sichuan provinces were 740.77, 327.28, and 185.37, respectively, ensuring the reliability of the posterior estimates, detailed results are presented in S1 Table. The pairwise posterior correlations among parameters suggest no significant identifiability issues (S4 Fig).

**Table 2:** The uniform distribution interval of the estimated parameter.

Parameter	Gansu	Guangdong	Sichuan
*a*	1 × 10^-9^ - 2 × 10^-9^	3 × 10^-8^ - 4 × 10^-8^	3 × 10^-9^ - 4 × 10^-9^
*b*	0 - 20	0 - 20	0 - 10
*c*	0 - 20	0 - 20	0 - 10
*a*_*h*_	1 × 10^-10^ - 3 × 10^-10^	2 × 10^-9^ - 3 × 10^-9^	9 × 10^-12^ - 2 × 10^-11^
*b*_*h*_	0 - 20	0 - 20	0 - 10
*c*_*h*_	0 - 20	0 - 20	0 - 10
*E*(0)	10,000 - 20,000	1,000 - 2,000	10,000 - 20,000
*I*(0)	30,000 - 40,000	2,000 - 4,000	20,000 - 40,000
*V*(0)	40,000 - 50,000	3,000 - 4,000	30,000 - 50,000

**Table 3:** Model parameter values.

Parameter	Gansu	Guangdong	Sichuan
*A*	808,426	28,142	406,571
*B*	17,770	90,495	48,972
$\mu_{\text{1}}$	0.00069	0.00046	0.00071
*a*	1.6920 × 10^-9^ (95%CI: 1.6624 × 10^-9^ - 1.7215 × 10^–9^)	3.6357 × 10^-8^ (95%CI: 3.6292 × 10^-8^ - 3.6422 × 10^–8^)	3.4040 × 10^-9^ (95%CI: 3.2441 × 10^-9^ - 3.5639 × 10^–9^)
*b*	10.1990 (95%CI: 9.4590 - 10.9390)	3.9255 (95%CI: 2.8731 - 4.9779)	2.8113 (95%CI: 1.4356 - 4.1870)
*c*	0.6767 (95%CI: 0.6094 - 0.7440)	1.7464 (95%CI: 1.5398 - 1.9530)	8.1169 (95%CI: 7.6462 - 8.5876)
*a*_*h*_	1.8350 × 10^-10^ (95%CI: 1.7149 × 10^-10^ - 1.9551 × 10^–10^)	2.5005 × 10^-10^ (95%CI: 2.2301 × 10^-10^ - 2.7709 × 10^–10^)	1.1895 × 10^-11^ (95%CI: 1.1769 × 10^-11^ - 1.2021 × 10^–11^)
*b*_*h*_	0.46715 (95%CI: 0.4199 - 0.5143)	0.6249 (95%CI: 0.5656 - 0.6842)	0.5017 (95%CI: 0.3991 - 0.6043)
*c*_*h*_	2.5752 (95%CI: 2.4648 - 2.6856)	10.9500 (95%CI: 10.7764 - 11.1236)	4.7185 (95%CI: 4.3757 - 5.0613)
*E*(0)	15,038 (95%CI: 12,130–17,946)	1,497 (95%CI: 1,209 - 1,785)	14,884 (95%CI: 12,073–17,695)
*I*(0)	35,081 (95%CI: 32,211–37,951)	3,006 (95%CI: 2,430–3,582)	30,063 (95%CI: 24,362–35,764)
*V*(0)	44,992 (95%CI: 42,084–47,900)	3,521 (95%CI: 3,233 - 3,809)	40,845 (95%CI: 35,276–46,414)

Setting initial values for each compartment constitutes the basis for model fitting. At the end of 2020, the total reported stock of sheep and cattle in Gansu province was *N*(0) = 26,738,000 [32]. To derive more reliable initial values for several uncertain parameters, we estimated *E*(0) = 15,038, *I*(0) = 35,081, and *V*(0) = 44,992 by calibrating the model to best fit new human brucellosis cases. The corresponding uniform prior distribution ranges and 95% confidence intervals for the estimates are shown in Tables 2 and 3, respectively. *S*(0) = *N*(0) - *V*(0) - *E*(0) - *I*(0) = 26,642,889. Since human brucellosis cases primarily occur in rural and pastoral areas, we obtained *S*_*h*_(0) = 11,952,499 based on the 2020 rural population of Gansu province [32]. Additionally, *S*_*ha*_(0) = 283 corresponds to the number of newly reported human brucellosis cases in January 2021. Finally, *S*_*hc*_(0) = *ω*_*h*_(1-*η*)*S*_*ha*_(0) = 44 can be derived. Similarly, we estimate the initial values for the brucellosis transmission model in Sichuan province as *N*(0) = 24,051,000, *E*(0) = 14,884, *I*(0) = 30,063, *V*(0) = 40,845, *S*(0) = 23,965,208, *S*_*h*_(0) = 36,208,956, *S*_*ha*_(0) = 12, *S*_*hc*_(0) = 2. Initial values for the brucellosis transmission model in Guangdong province as *N*(0) = 2,166,996, *E*(0) = 1497, *I*(0) = 3006, *V*(0) = 3521, *S*(0) = 2,158,972, *S*_*h*_(0) = 32,576,436, *S*_*ha*_(0) = 30, *S*_*hc*_(0) = 5. To present the specific numerical values more intuitively, this study summarizes the dataset spans, initial values, and parameter values used in the provincial models, as shown in S2 Table.

### 3.3. Model fitting

**Fig 4:**
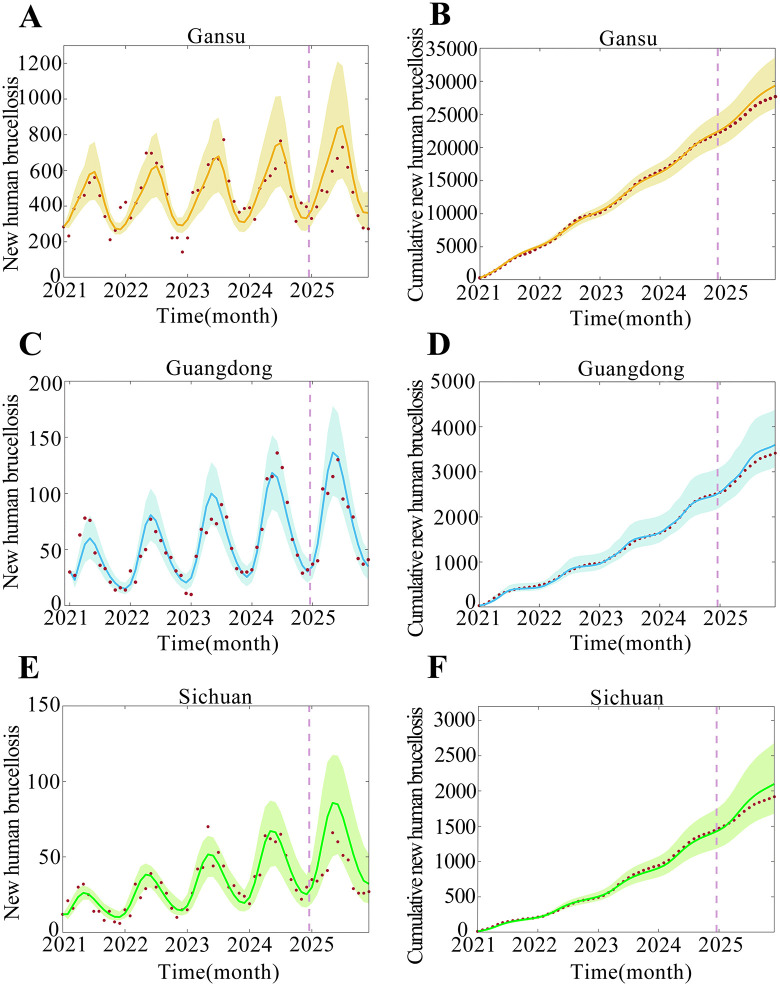
The model separately fitted the trends of new human brucellosis cases in Gansu, Guangdong, and Sichuan provinces from 2021 to 2024, and predicted the trends for the year 2025. **(A)**, **(C)** and **(E)** The new human brucellosis cases in Gansu, Guangdong and Sichuan provinces, respectively. **(B)**, **(D)** and **(F)** The cumulative new human brucellosis cases in Gansu, Guangdong and Sichuan provinces, respectively. Reddish-brown solid dots indicate actual values, while orange, blue, and green curves represent the model-fitted values for Gansu, Guangdong and Sichuan provinces, respectively. The light-colored areas around the curves denote the 95% confidence intervals of the fitted curves.

**Fig 5:**
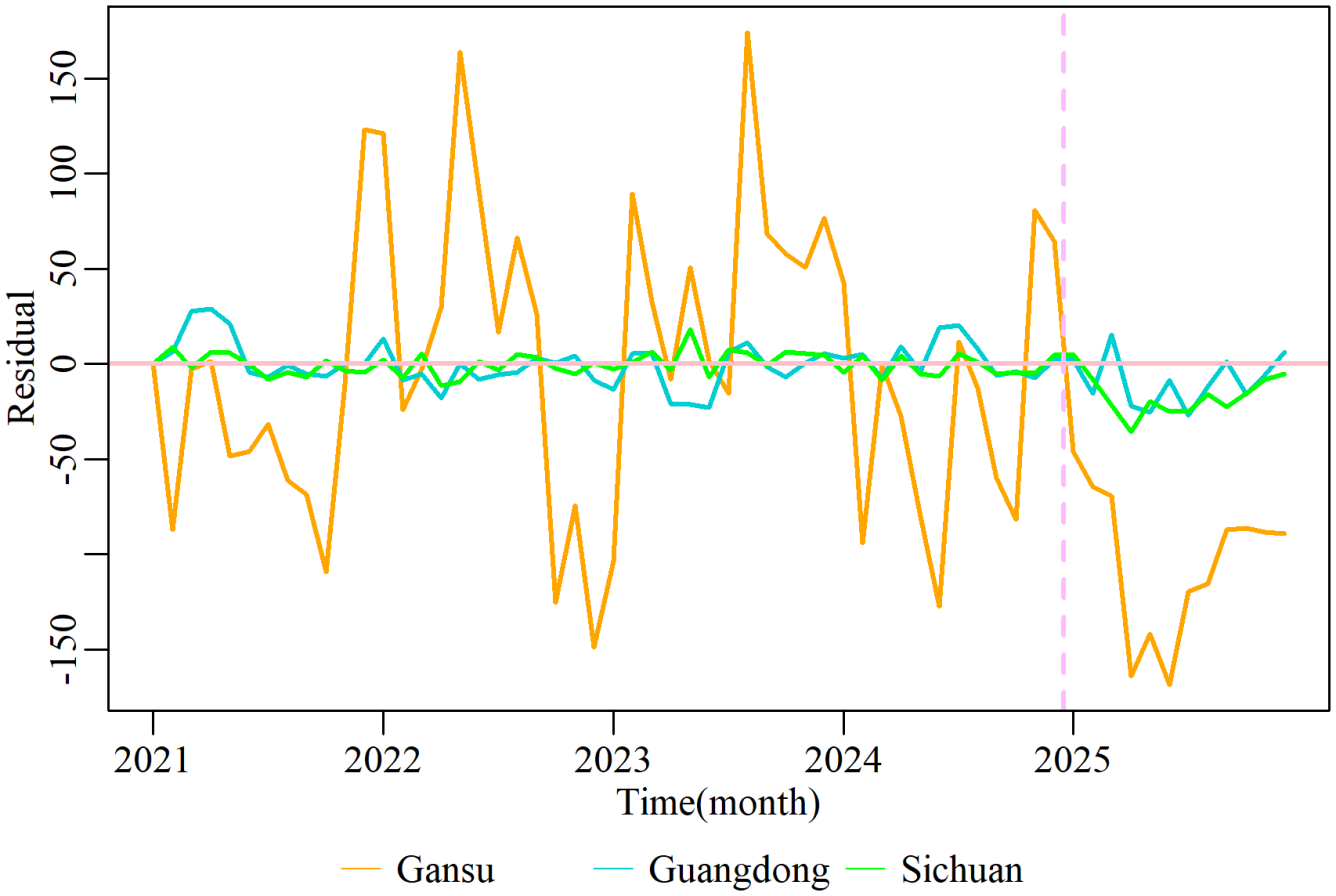
Residual plots of observed versus model-predicted values in Gansu, Guangdong, and Sichuan provinces.


The developed seasonal dynamic model separately fitted the trends in monthly new and cumulative new human brucellosis cases in Gansu, Guangdong and Sichuan provinces from 2021 to 2024, as shown in Fig 4. The model-fitted values align well with the observed values, and almost all data points lie within the 95% confidence interval of the fitted curve. Fig 5 presents the residuals between the observed values and the predictions of the seasonal dynamic model for Gansu, Guangdong and Sichuan provinces. Specifically, the number of newly reported human brucellosis cases declined slightly in 2025, which led to marginally higher model-predicted values compared with the observed values. The mean absolute percentage error (*MAPE*) was used to evaluate the model fitting accuracy [37]. The formula is expressed as

$$\begin{array}{c} MAPE=\left(\frac{\text{1}}{n}\sum_{t=1}^{n}\frac{W_{t}-Q_{t}}{W_{t}}\right)\times 100\%\text{,} \end{array}$$

where *W*_*t*_ represents the observed number of new human brucellosis cases at time *t*, *Q*_*t*_ denotes the model-fitted value at time *t*, and *n* is the sample size of the data used for prediction. The *MAPE* values of the model-fitted new human brucellosis cases for Gansu, Guangdong and Sichuan provinces were 16.58%, 20.27% and 21.39%, respectively. For cumulative new human brucellosis cases, the *MAPE* values were 2.47%, 3.58%, and 4.52%, respectively. Detailed *MAPE* results are presented in Table 4. The results indicate that the deviation between the model predictions and the observed values is within a reasonable range. The established seasonal SEIV dynamic model can satisfactorily fits the fluctuating trends of new human brucellosis cases and is well consistent with the actual epidemic incidence of human brucellosis in Gansu, Guangdong, and Sichuan provinces.

**Table 4:** The *MAPE* values for model simulation and prediction.

Model	Time	Cases	Gansu	Guangdong	Sichuan
Seasonal SEIV	2021 - 2024	New cases	16.58%	20.27%	21.39%
	2025	New cases	22.56%	17.06%	24.59%
	Total	New cases	17.89%	19.32%	22.42%
Seasonal SEIV	2021 - 2023	New cases	16.03%	23.43%	22.51%
	2024	New cases	25.77%	23.15%	24.24%
	Total	New cases	18.47%	23.36%	22.94%
SARIMA	2021 - 2024	New cases	15.12%	20.45%	23.84%
	2025	New cases	19.86%	18.51%	20.11%
	Total	New cases	16.47%	19.44%	23.09%
Seasonal SEIV	2021 - 2024	Cumulative new cases	2.47%	3.58%	4.52%
	2025	Cumulative new cases	3.49%	3.61%	5.13%
	Total	Cumulative new cases	2.67%	3.59%	4.64%

To validate the simulation and predictive performance of the seasonal SEIV model, we constructed the model using data on new human brucellosis cases from 2021 to 2023 to predict the cases in 2024. The simulation results are presented in S5 Fig, and the *MAPE* values are listed in Table 4. At the same time, we constructed Seasonal Autoregressive Integrated Moving Average (SARIMA) models to simulate and predict new human brucellosis cases in Gansu, Guangdong, and Sichuan provinces, calculating corresponding *MAPE* values of 15.12%, 20.45%, and 23.84%, respectively. The SARIMA model construction and detailed results are presented in S1 File. Overall, the seasonal SEIV dynamic model showed a slightly better fitting performance than the SARIMA model, and it can predict the long-term trends of new human brucellosis cases, thus providing a quantitative basis for the scientific formulation of effective prevention and control measures.

### 3.4. Model prediction

**Fig 6:**
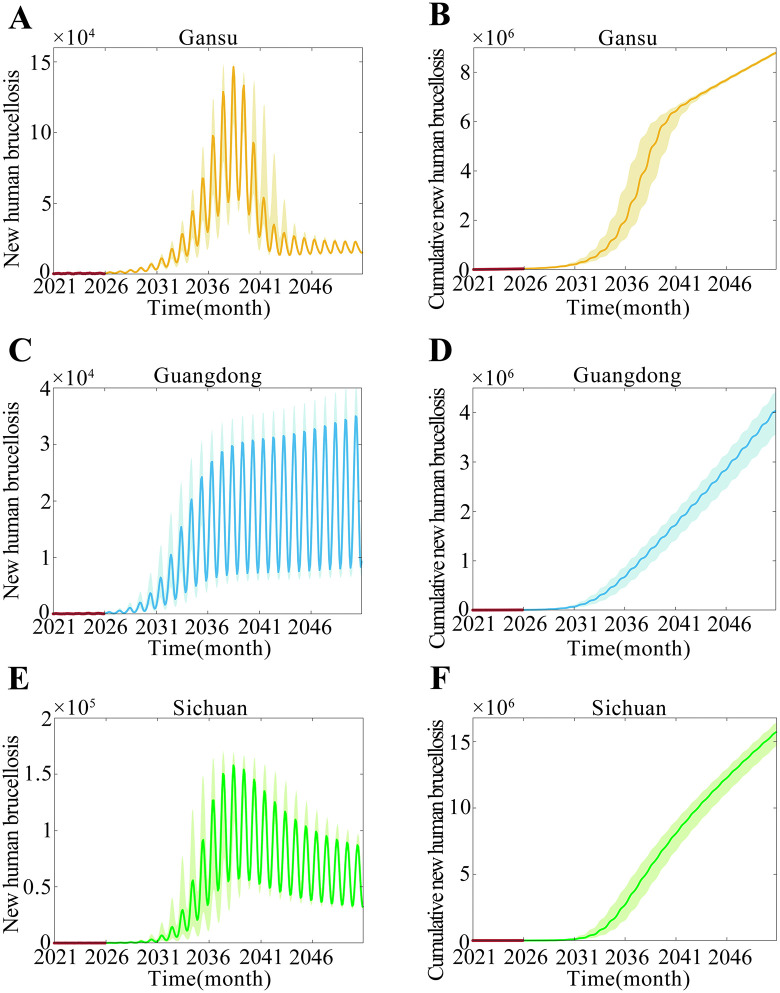
The model predicts the trends of new and cumulative new human brucellosis cases in Gansu, Guangdong and Sichuan provinces from 2025 to 2050. **(A)**, **(C)** and **(E)** The number of new human brucellosis cases in Gansu, Guangdong and Sichuan provinces, respectively. **(B)**, **(D)** and **(F)** The number of cumulative new human brucellosis cases in Gansu, Guangdong and Sichuan provinces, respectively. Reddish-brown solid dots indicate actual values, while orange, blue and green curves represent the model-fitted values for Gansu, Guangdong and Sichuan provinces, respectively. The light-colored areas around the curves denote the 95% confidence intervals of the fitted curves.

Based on the model’s good fitting performance, the fluctuation trends in the number of new and cumulative new human brucellosis cases in Gansu, Guangdong and Sichuan provinces from 2025 to 2050 were predicted separately, as shown in Fig 6. The short-term prediction from January to October 2025 showed that the model-predicted values were in good agreement with the actual values, almost within the 95% confidence interval of the predicted value, as shown in Fig 5. Long-term predictions indicate that the number of new human brucellosis cases in Gansu and Sichuan provinces will first gradually increase, reaching peak values of 146,310 (95%CI: 126,845–165,775) and 157,903 (95%CI: 126,700–189,106) cases in July 2038 and May 2038, respectively. Subsequently, these numbers will gradually decrease to a stable state and continue to epidemic. The number of new human brucellosis cases in Guangdong province will gradually increase and reach a relatively stable state in 2038. The model prediction results are shown in Table 5.

**Table 5:** Model-predicted values for new human brucellosis cases during the peak incidence periods in Gansu, Guangdong and Sichuan provinces.

Year	Month	Area		
		Gansu	Guangdong	Sichuan
2038	1	55,061 (95%CI: 43,445–66,677)	10,903 (95%CI: 5,361–16,445)	69,947 (95%CI: 41,236–98,658)
	2	68,326 (95%CI: 53,109–83,543)	15,641 (95%CI: 8,809–22,473)	92,913 (95%CI: 56,153–129,673)
	3	89,722 (95%CI: 70,548–108,896)	21,248 (95%CI: 14,433–28,063)	120,623 (95%CI: 79,466–161,780)
	4	115,316 (95%CI: 93,014–137,618)	26,499 (95%CI: 20,345–32,654)	145,002 (95%CI: 104,728–185,277)
	5	137,177 (95%CI: 114,516–159,839)	29,730 (95%CI: 24,553–34,907)	157,903 (95%CI: 126,700–189,106)
	6	138,668 (95%CI: 117,411–159,925)	29,573 (95%CI: 24,444–34,702)	155,007 (95%CI: 125,952–184,063)
	7	146,310 (95%CI: 126,845–165,775)	25,851 (95%CI: 19,578–32,125)	137,729 (95%CI: 111,116–164,343)
	8	118,158 (95%CI: 102,000–134,316)	19,811 (95%CI: 12,427–27,196)	112,235 (95%CI: 86,790–137,681)
	9	93,300 (95%CI: 80,338–106,263)	13,524 (95%CI: 6,871–20,177)	86,438 (95%CI: 64,355–108,522)
	10	71,737 (95%CI: 62,050–81,425)	8,933 (95%CI: 4,643–13,223)	66,838 (95%CI: 49,728–83,948)
	11	57,648 (95%CI: 50,440–64,857)	7,088 (95%CI: 5,157–9,020)	57,119 (95%CI: 45,252–68,987)
	12	52,422 (95%CI: 47,033–57,811)	8,032 (95%CI: 4,506–11,558)	58,720 (95%CI: 44,170–73,270)

**Fig 7:**
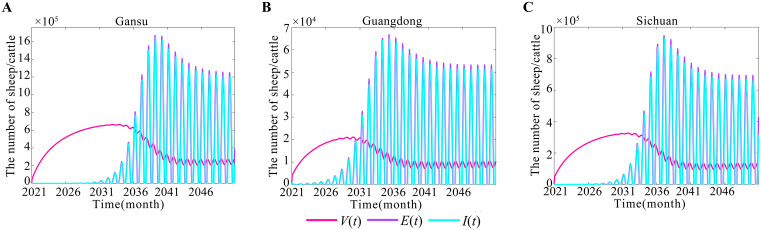
The model predicts trends for various sheep/cattle subgroups in Gansu, Guangdong, and Sichuan provinces from 2021 to 2050. **(A)** Trends in infected, exposed, and vaccinated sheep/cattle in Gansu province. **(B)** Trends in infected, exposed, and vaccinated sheep/cattle in Guangdong province. **(C)** Trends in infected, exposed, and vaccinated sheep/cattle in Sichuan province.

We separately projected the trends in infected *I*(*t*), exposed *E*(*t*), and vaccinated *V*(*t*) sheep/cattle in Gansu, Guangdong, and Sichuan provinces from 2021 to 2050, as shown in Fig 7. The number of exposed and infected sheep/cattle both showed a rapid increase, followed by a gradual decline toward a stable state. The number of vaccinated sheep/cattle showed an inverse trend compared to the number of exposed and infected sheep/cattle, ultimately stabilizing at a lower level. The peaks in the number of exposed and infected sheep/cattle in Gansu, Guangdong, and Sichuan provinces occurred in 2038, 2036, and 2037, respectively. These peaks all appeared either before or in the same year as the peak in new human brucellosis cases in 2038, consistent with the temporal relationship of transmission from sheep/cattle to humans.

### 3.5. Estimation of the basic reproduction number *R*_0_

Using the method of Wang et al. [29] for calculating the basic reproduction number *R*_0_ with periodic parameters, the estimated *R*_0_ for brucellosis transmission in Gansu, Guangdong and Sichuan provinces were 2.2510 (95%CI: 2.2160-2.2859), 2.7937 (95%CI: 2.7592-2.8283) and 2.9499 (95%CI: 2.9007-2.9992), respectively, as shown in Table 6. The results indicate that under current prevention and control measures, human brucellosis will continue to be endemic in Gansu, Guangdong and Sichuan provinces and cannot be eliminated yet. The method of van den Driessche and Watmough [30] can be used to calculate the average basic reproduction number ${\tilde{\text{R}}}_{\text{0}}$, and the formula is

**Table 6:** The values of ${𝐑}_{0}$ and ${\tilde{𝐑}}_{0}$ for brucellosis transmission in Gansu, Guangdong and Sichuan provinces.

Area	Basic reproduction number $R_{0}$	Average basic reproduction number ${\tilde{R}}_{0}$
Gansu	2.2510 (95%CI: 2.2160-2.2859)	2.2518 (95%CI: 2.2168-2.2868)
Guangdong	2.7937 (95%CI: 2.7592-2.8283)	2.7951 (95%CI: 2.7605-2.8295)
Sichuan	2.9499 (95%CI: 2.9007-2.9992)	2.9511 (95%CI: 2.9016-3.0001)


$$\begin{array}{c} {\tilde{R}}_{\text{0}}=\frac{\overset{―}{\beta }A\text{(}f+\lambda +\mu \text{)(}\delta +\mu +\varepsilon v\text{)}}{\mu \text{(}f+\mu \text{)(}\lambda +\mu \text{)(}\delta +\mu +v\text{)}}. \end{array}$$

**Fig 8:**
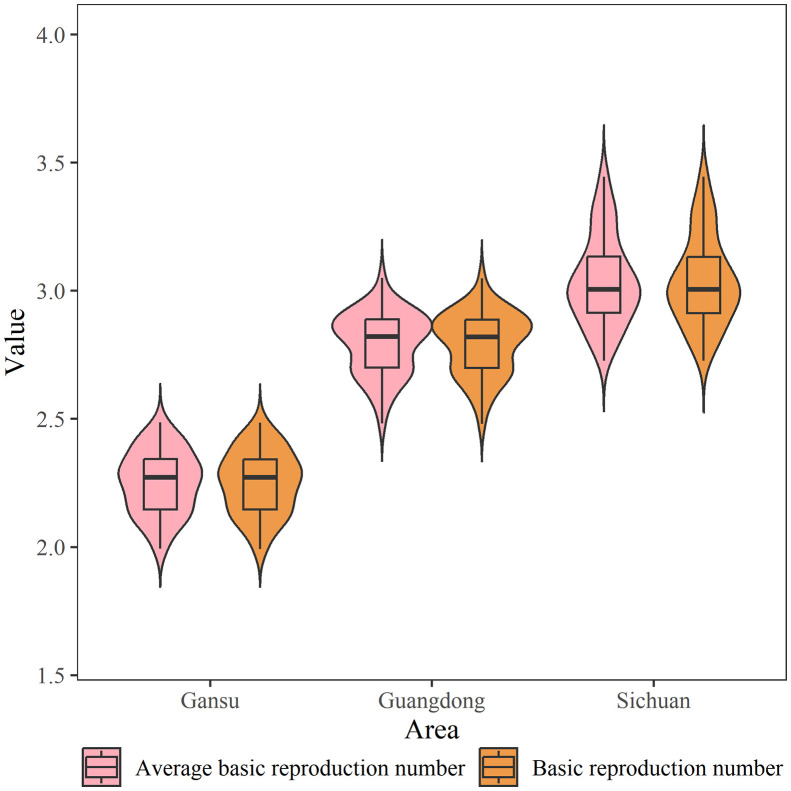
Violin plots of the basic reproduction number *R*_0_ and the average basic reproduction number ${\tilde{R}}_{0}$.

In this study, the estimated ${\tilde{\text{R}}}_{\text{0}}$ for brucellosis transmission in Gansu, Guangdong and Sichuan provinces were 2.2518 (95%CI: 2.2168-2.2868), 2.7951 (95%CI: 2.7605-2.8295), and 2.9511 (95%CI: 2.9016-3.0001), respectively, all slightly higher than the *R*_0_ value. This implies that calculating ${\tilde{R}}_{\text{0}}$ using the mean value method slightly overestimates the intensity of brucellosis transmission. Additionally, we plotted violin plots of the estimated values ${\text{R}}_{\text{0}}$ and ${\tilde{\text{R}}}_{\text{0}}$for comparison, as shown in Fig 8.

### 3.6. Model parameter Screening

**Fig 9:**
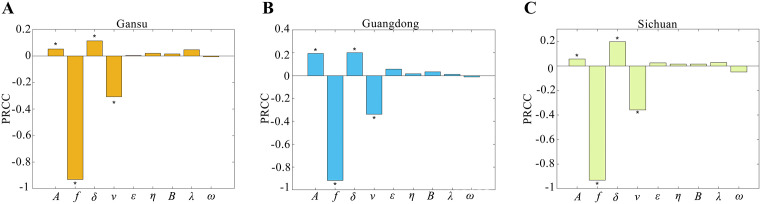
PRCC results for the dependence of new human brucellosis cases on each parameter. **(A)** Gansu province. **(B)** Guangdong province. **(C)** Sichuan province. * indicates *P* < 0.05, suggesting that the parameter has a significant influence.

A sensitivity analysis based on Partial Rank Correlation Coefficients (PRCC) was employed to evaluate the impact of each parameter on the number of new human brucellosis cases, and all PRCC values were calculated using Latin Hypercube Sampling (LHS) [38]. The larger the absolute value of the PRCC, the more significantly the parameter affects fluctuations in the number of new human brucellosis cases. We choose the sample size *n* = 1000, with the model parameters as the input variables, and the number of new human brucellosis cases as the output variable. All parameters were assumed to follow a uniform distribution, with a significance level set at *α* = 0.05, a parameter was considered statistically significant if its *P* value was less than 0.05. The exact PRCC values and *P* values for each parameter are presented in Table 7. Fig 9 depicts the PRCC values of each parameter, with parameters marked by an asterisk above the bars being statistically significant. Therefore, we can clearly identify the model parameters with significant influence as *f*, *ν*, *δ*, and *A*; specifically, parameters *A* and *δ* exert a positive impact on the number of new human brucellosis cases, whereas *f* and *ν* have negative impact.

**Table 7:** Partial rank correlation coefficients between new human brucellosis cases and each input parameter variable.

Input parameter	Gansu		Guangdong		Sichuan	
	PRCC	*P* value	PRCC	*P* value	PRCC	*P* value
*A*	0.0546	0.0473	0.1935	9.0083 × 10^-11^	0.0562	0.0469
*f*	-0.9308	0	-0.9166	0	-0.9317	0
*δ*	0.1148	3.0502 × 10^-5^	0.2008	1.9920 × 10^-11^	0.1977	3.7824 × 10^-11^
*ν*	-0.3073	5.2886 × 10^-24^	-0.3382	8.4211 × 10^-29^	-0.3582	3.2397 × 10^-31^
*ε*	0.0035	0.9125	0.0506	0.0758	0.0252	0.4288
*η*	0.0198	0.5334	0.0161	0.6125	0.0149	0.6409
*B*	0.0157	0.6231	0.0335	0.2935	0.0153	0.6309
*λ*	0.0467	0.1431	0.0106	0.7385	0.0270	0.3969
*ω*	-0.0054	0.8655	-0.0110	0.7298	-0.0484	0.1288

### 3.7. Parameter value analysis for key parameters *f* and *v*

**Fig 10:**
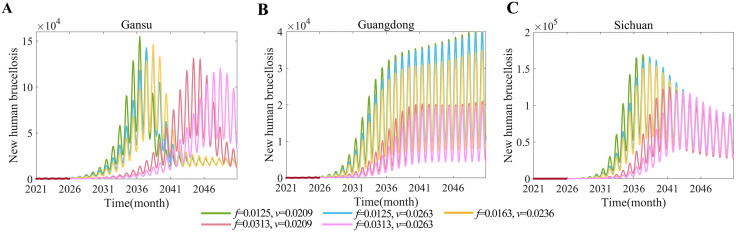
The effects of variations in parameters *f* and *v* on new human brucellosis cases in three provinces. **(A)** Gansu province. **(B)** Guangdong province. **(C)** Sichuan province.

Previous studies reported that the culling rate of infected sheep/cattle *f* ranged from 0.0125 to 0.0313, and the vaccination rate of susceptible sheep/cattle *v* ranged from 0.0209 to 0.0263 across several provinces in China. In this study, we used the provincial mean values *f* = 0.0163 and *v* = 0.0236 for model analysis. Among the possible control combinations, *f* = 0.0125 and *v* = 0.0209 represented the lowest control level, while *f* = 0.0313 and *v* = 0.0263 represented a relatively stringent control level. However, no provinces have been reported to adopt either extreme. Our parameter values fell between the reported control levels (*f* = 0.0125, *v* = 0.0236) and (*f* = 0.0313, *v* = 0.0209), and were closer to the current lower control level. The effects of variations in *f* and *v* on new human brucellosis cases in Gansu, Guangdong, and Sichuan provinces are presented in Fig 10. Compared with *v*, parameter *f* had a greater impact on new human brucellosis cases and was more effective in controlling disease transmission. Thus, based on the selected values of *f* and *v*, the proposed control measures achieve an ideal level that reasonably covers the epidemic in Chinese provinces and effectively controls brucellosis transmission.

### 3.8. Sensitivity analysis of parameters to the number of new human brucellosis cases

**Fig 11:**
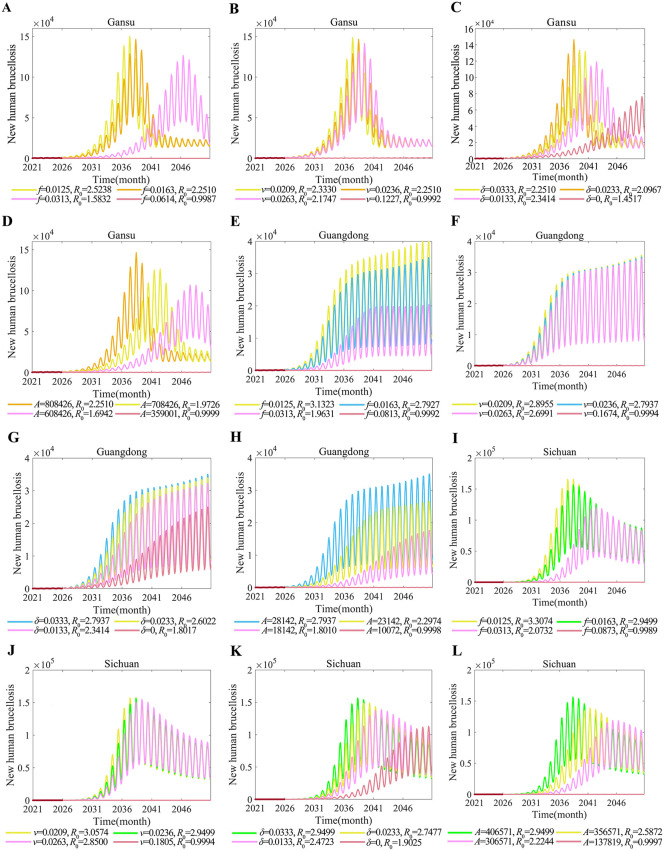
The effects of parameters variations on new human brucellosis cases in Gansu, Guangdong, and Sichuan provinces. **(A)**, **(B)**, **(C)** and **(D)** The effects of *f*, *v*, *δ* and *A* on new human brucellosis cases in Gansu province, respectively. **(E)**, **(F)**, **(G)** and **(H)** The effects of *f*, *v*, *δ* and *A* on new human brucellosis cases in Guangdong province, respectively. **(I)**, **(J)**, (K) and **(L)** The effects of *f*, *v*, *δ* and *A* on new human brucellosis cases in Sichuan province, respectively.

The effects of parameter changes on the number of new human brucellosis cases in Gansu, Guangdong and Sichuan provinces are shown in Fig 11, respectively. As the culling rate of infected sheep/cattle *f* increases, the peak number of new human brucellosis cases gradually decreases, indicating a reduction in cases and a delay in the timing of the peak. When *f* in Gansu, Guangdong and Sichuan provinces exceeds 0.0613, 0.0812 and 0.0872, respectively, the number of new human brucellosis cases gradually approaches zero, indicating that brucellosis is effectively controlled and eventually eliminated, as shown in Fig 11A, 11E and 11I. Similarly, as the vaccination rate of susceptible sheep/cattle *v* increases, the peak number of corresponding new human brucellosis cases decreases and is delayed. When *v* in Gansu, Guangdong and Sichuan provinces exceeds 0.1226, 0.1673, and 0.1804, respectively, brucellosis transmission is effectively suppressed, and the number of new human cases gradually approaches zero, as shown in Fig 11B, 11F and 11J. As the immune loss rate of vaccinated sheep/cattle *δ* decreases, the number of new human brucellosis cases in all three provinces shows a gradual downward trend, with the occurrence of the peak is also delayed. However, even when *δ* is reduced to zero, new human brucellosis cases remain endemic and cannot be completely controlled, as shown in Fig 11C, 11G and 11K. The new recruitment of sheep/cattle *A* in Gansu, Guangdong, and Sichuan provinces has been reduced to 359,002, 10,073, and 137,820 respectively, which can also control the spread of brucellosis, as shown in Fig 11D, 11H and 11L. Meanwhile, *R*_0_ was confirmed as a threshold for determining whether new human brucellosis cases are endemic. A larger *R*_0_ is associated with a higher number of new human cases and an earlier timing of the peak. When *R*_0_ > 1, new human brucellosis cases exhibit an epidemic trend and gradually stabilize into sustained transmission. Conversely, when *R*_0_ < 1, human brucellosis can be effectively controlled and gradually approach zero.

Simultaneously, we analyzed the effects of parameters *f, v, δ* and *A* on infected, exposed, and vaccinated sheep/cattle, respectively. As *f* and *v* increase, or *δ* and *A* decrease, the peak values reached by infected and exposed sheep/cattle decrease, and the timing is delayed, as shown in S9 and S10 Figs, respectively. Conversely, as *f* and *v* increase, the number of vaccinated sheep/cattle increases; as *δ* and *A* increase, the number of vaccinated sheep/cattle first gradually increases and then decreases to a steady state, as shown in S11 Fig. Currently, as *f* and *v* increase, or *A* decreases, it is possible to reduce the number of infected, exposed, and vaccinated sheep/cattle to zero individually. However, even if *δ* is reduced to zero, the number of infected, exposed, or vaccinated sheep/cattle will still gradually increase, leading to a widespread outbreak.

### 3.9. Sensitivity analysis of parameters to *R*_0_

**Fig 12:**
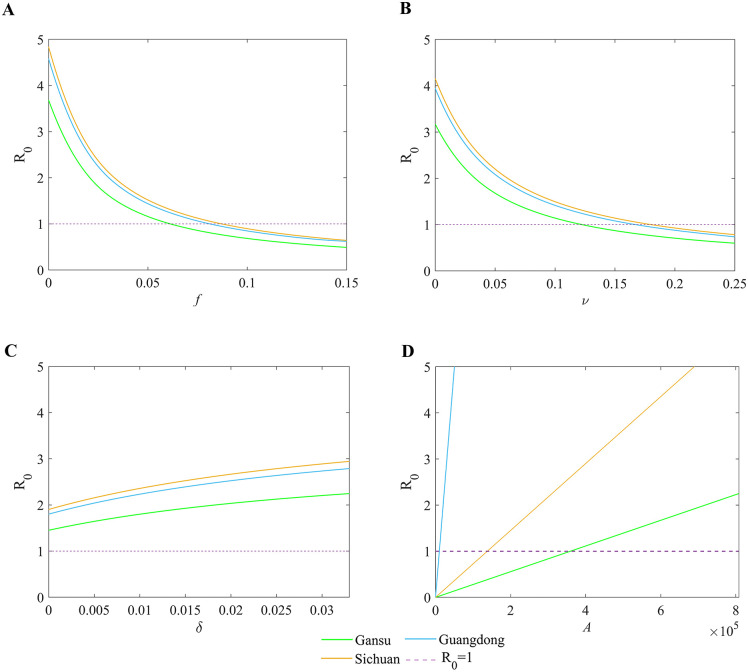
The effects of parameters variations on the *R*_0_ of brucellosis transmission in Gansu, Guangdong and Sichuan provinces. (A) ***f*.** (B) ***v*.** (C) *δ*. **(D)** *A.*

By analyzing the impact of model parameter changes on the basic reproduction number *R*_0_, effective prevention and control measures can be proposed. The effects of changes in parameters *f*, *v*, *δ* and *A* on *R*_0_ are shown in Fig 12. As the culling rate of infected sheep/cattle *f* increases, *R*_0_ gradually decreases and can become less than 1. When *f* in Gansu, Guangdong and Sichuan provinces exceeds 0.0613, 0.0812 and 0.0872, respectively, can make *R*_0_ < 1, indicating that brucellosis is effectively controlled and eventually eliminated, as shown in Fig 12A. Increasing the vaccination rate of susceptible sheep/cattle *v* also reduces *R*_0_. When *v* in Gansu, Guangdong and Sichuan provinces overrun 0.1226, 0.1673 and 0.1804, respectively, can make *R*_0_ < 1, as shown in Fig 12B. Decreasing the immune loss rate of vaccinated sheep/cattle *δ* slightly reduces *R*_0_, but cannot make *R*_0_ < 1. It can be implemented in conjunction with other control measures, as shown in Fig 12C. Reduce the new recruitment of sheep/cattle *A* in Gansu, Guangdong, and Sichuan provinces to levels below 359,002, 100,730, and 137,820, respectively, to achieve *R*_0_ < 1 and progressively eradicate brucellosis, as shown in Fig 12D. Increasing the culling rate of infected sheep/cattle *f* and the vaccination rate for susceptible sheep/cattle *v*, while decreasing the immune loss rate of vaccinated sheep/cattle *δ* and the new recruitment of sheep/cattle *A* are effective measures for controlling human brucellosis in Gansu, Guangdong and Sichuan provinces.

### 3.10. Control strategy analysis

**Fig 13:**
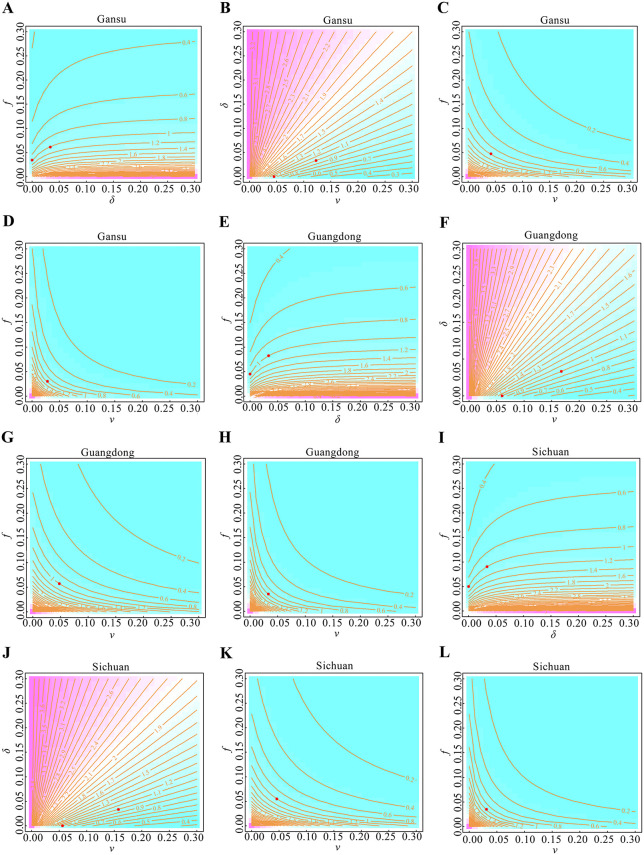
Contour plots of *R*_0_ on *f*, *v* and *δ* in Gansu, Guangdong and Sichuan Provinces. **(A)**, **(B)**, **(C)** and **(D)** The impact of *f* and *δ*, *v* and *δ*, *f* and *v*, *f*, *v* and *δ* on *R*_0_ in Gansu province, respectively. **(E)**, **(F)**, **(G)** and **(H)** The impact of *f* and *δ*, *v* and *δ*, *f* and *v*, *f*, *v* and *δ* on *R*_0_ in Guangdong province, respectively. **(I)**, **(J)**, **(K)** and **(L)** The impact of *f* and *δ*, *v* and *δ*, *f* and *v*, *f*, *v* and *δ* on *R*_0_ in Sichuan province, respectively. The orange curve represents the *R*_0_ contour lines. The red dot indicates the threshold for the selected control measure.

Reducing the new recruitment of sheep/cattle *A* to control brucellosis transmission would severely impact herders’ economic income and the market supply of mutton and beef. Therefore, we primarily consider the combined implementation of *f*, *v*, and *δ* to eliminate brucellosis. Following vaccination, immune protection in sheep/cattle gradually diminishes over time, resulting in an immune loss rate of 0.0333. By establishing uniform vaccine selection criteria and immunization protocols, strengthening post-vaccination antibody monitoring, and implementing timely booster vaccinations [39], the immune loss rate can be reduced from the current 0.0333 to 0. In Gansu province, when *δ* = 0, either *f* > 0.0351 or *v* > 0.0452 can achieve *R*_0_ < 1. This indicates that *δ* combined with either *f* or *v* can control brucellosis transmission, as shown in Fig 13A and 13B. We found multiple combinations of *f* and *v* that yield *R*_0_ < 1. We select the point on the *R*_0_ = 1 contour line closest to the origin (0, 0) as the optimal combined measure for *f* and *v*. At this point, the recommended thresholds for control measures are relatively small, making the resulting public health decisions easier to implement. Therefore, when *δ* = 0.0333, *f* > 0.0471 and *v* > 0.0420, can achieve *R*_0_ < 1, indicating that the combined control of *f* and *v* can effectively eliminate brucellosis, as shown in Fig 13C. Finally, we analyzed the combined effects of implementing *f*, *v* and *δ*. When *δ* = 0, *f* > 0.0303 and *v* > 0.0285, *R*_0_ < 1 can be achieved, indicating that the combined implementation of all measures is the optimal strategy for controlling brucellosis transmission, results are shown in Fig 13D. Similarly, we analyzed the effectiveness of combined implementation of *f*, *v* and *δ* in controlling brucellosis in Guangdong province, the results are shown in Fig 13E, 13F, and 13G, respectively. The final optimal control measures identified are *δ =* 0, *f* > 0.0356 and *v* > 0.0314, as shown in Fig 13H. The effects of combined implementation of *f*, *v* and *δ* on *R*_0_ in Sichuan province are presented in Fig 13I, 13J, and 13K. The recommended optimal control measures are *δ =* 0, *f* > 0.0358, *v* > 0.0335, as shown in Fig 13L. Additionally, we detail the recommended threshold values for combined brucellosis prevention and control strategies in Gansu, Guangdong, and Sichuan provinces, as presented in Table 8.

**Table 8:** Recommended thresholds for joint measures to control brucellosis transmission in Gansu, Guangdong, and Sichuan provinces.

Control measures	Threshold for control measures			Effect evaluation
	Gansu	Guangdong	Sichuan	
δ and v	δ = 0.0333, v > 0.1226; δ = 0, v > 0.0452.	δ = 0.0333, v > 0.1673; δ = 0, v > 0.0602.	δ = 0.0333, v > 0.1804; δ = 0, v > 0.0606.	Effective Measures
δ and f	δ = 0.0333, f > 0.0613; δ = 0, f > 0.0351.	δ = 0.0333, f > 0.0812; δ = 0, f > 0.0457.	δ = 0.0333, f > 0.0872; δ = 0, f > 0.0503.	Effective Measures
v and f	v > 0.0420, f > 0.0471	v > 0.0463, f > 0.0550	v > 0.0485, f > 0.0572	Effective Measures
δ, v and f	δ = 0, f > 0.0303, v > 0.0285	δ = 0, f > 0.0356, v > 0.0314	δ = 0, f > 0.0358, v > 0.0335	Optimal Measures

## 4. Discussion

As a prevalent zoonotic disease, brucellosis has caused triggered a host of non-negligible economic losses and public health burdens globally, and it also ranks among the major public health challenges in China [40]. The Chinese Center for Disease Control and Prevention is actively exploring effective and practical strategies for the prevention and control of brucellosis transmission. A wide range of prevention and control strategies have been proposed by researchers, such as screening infected animals via detection techniques, implementing vaccination for susceptible animals, culling infected animals, and performing environmental disinfection [3].

In this study, the constructed seasonal SEIV dynamic model was applied to fit the new human brucellosis cases in Gansu, Guangdong and Sichuan provinces during 2021–2024, with model parameters estimated via NLS and MCMC methods. The simulation results exhibited a good consistency with the newly reported human brucellosis cases. The basic reproduction numbers *R*_0_ of brucellosis transmission was estimated to be 2.2510, 2.7937 and 2.9499 for Gansu, Guangdong and Sichuan provinces, respectively, indicating that the disease remains highly prevalent. It is predicted that the number of new human brucellosis cases will keep increasing over the next decade. Furthermore, we explored the effects of various intervention strategies through parameter sensitivity analysis. The results revealed that raising the vaccination rate of susceptible sheep/cattle, increasing the culling rate of infected sheep/cattle, and simultaneously reducing the immune loss rate of vaccinated sheep/cattle can effectively and sustainably curb brucellosis transmission. The brucellosis prevention and control measures proposed in this study are consistent with previous research the findings [28,34,36].

Dynamic models serve as essential tools for dissecting the complex transmission mechanisms of brucellosis, capturing the interactions among infectious sources, transmission pathways, susceptible populations, as well as natural and social environmental factors. Under the current prevention and control measures, the number of new human brucellosis cases in Gansu, Guangdong, and Sichuan provinces exhibit a distinct upward trend. Our model analysis suggests that this trend may stem from the combined effects of the following multifaceted mechanisms: (1) The culling rate of infected sheep/cattle remains low. Currently, the culling rate of infected sheep/cattle is only 0.0163. Under optimal control strategies, the threshold levels to be achieved in Gansu, Guangdong, and Sichuan provinces are 0.0303, 0.0356, and 0.0358, respectively, indicating that a significant gap still exists. A low culling rate of infected sheep/cattle leads to persistently high infection rates among livestock, thereby maintaining the risk of human brucellosis transmission. (2) Insufficient vaccination rate. The current vaccination rate for susceptible sheep/cattle stands is 0.0236, while Gansu, Guangdong, and Sichuan provinces need to increase their rates to 0.0285, 0.0314, and 0.0335, respectively. Inadequate vaccination rates among sheep/cattle will heighten the overall susceptibility of livestock population, consequently elevating the risk of human infection with brucellosis. (3) Relatively high immunity loss rate in vaccinated sheep/cattle. The vaccine’s efficacy gradually declines, leading to an immunity loss rate of 0.0333 in vaccinated sheep/cattle. These previously protected animals revert to a susceptible state, thereby increasing the number of infected sheep/cattle. (4) Seasonal factors. Brucellosis exhibits distinct seasonal characteristics, with summer and autumn being peak periods. This phenomenon is associated with animal breeding activities, peak miscarriages period, and increased bacterial shedding during lactation, all of which raise the infection risk in both sheep/cattle and humans. In addition to the factors examined in this study, other contributing elements may include infrequent environmental disinfection and inadequate quarantine of sheep/cattle [41].

Limiting the new recruitment of sheep/cattle can curb brucellosis transmission, yet it hinders livestock industry development and meat supply, seriously affecting residents’ income [42]. This measure is of low feasibility. Therefore, Consistent with the views of other scholars, this study excludes limiting the new recruitment of sheep/cattle from brucellosis control strategies. Instead, raising the culling rate of infected sheep/cattle and the vaccination rate of susceptible sheep/cattle are the primary measures for controlling the spread of brucellosis. Hou et al. [19] and Liu et al. [33] argue that relying on a single control measure alone is insufficient to eliminate brucellosis, combined implementation of multiple strategies is essential. We also find that relying solely on either raising the culling rate of infected sheep/cattle or increasing the vaccination rate of susceptible sheep/cattle requires unrealistically high implementation levels, potentially even above 100%. Therefore, we conclude that integrated control measures are feasible. Within the scope of implementable prevention policies, it is possible to identify relatively low levels for both the culling rate of infected sheep/cattle and the vaccination rate of susceptible sheep/cattle to achieve the recommended threshold of control efficacy. The immune protection conferred by vaccination in sheep/cattle is not permanent. Studies have shown that antibody levels remain high in the early stage after vaccination, but gradually decline over time, thereby weakening the immune protective effect [39]. Currently, China primarily vaccinates sheep/cattle with the B. suis strain 2 (S2) vaccine, its protective efficacy reaches 68.5% at 12 months post-vaccination, and drops to 46.7% by 24 months post-vaccination [43]. Many studies have not considered reducing the immune loss rates in vaccinated sheep/cattle as a measure to control brucellosis transmission. This may be attributed to the fact that its efficacy can not be significantly reflected by increasing the vaccination rate of susceptible sheep/cattle or the culling rate of infected sheep/cattle, as well as it requires significant investment [44]. Of course, this study also demonstrates that even reducing immune loss rates to zero cannot effectively contain brucellosis transmission by this single intervention alone. Establishing uniform vaccine selection criteria and standardized immunization protocols, promoting vaccines with stable immunogenicity and prolonged protective efficacy (such as the M5-90Δ26 strain vaccine), and conducting regular antibody surveillance and timely booster vaccinations for vaccinated sheep/cattle are feasible approaches to reduce immune loss rates to zero [39]. This becomes an effective supplementary measure to curb the spread of brucellosis. Ultimately, this study concludes that the optimal brucellosis prevention and control strategy for Gansu, Guangdong, and Sichuan provinces lies in the combined implementation of three interventions: raising the culling rate of infected sheep/cattle, increasing vaccination rate of susceptible sheep/cattle, and lowing the immune loss rate of vaccinated sheep/cattle. Naturally, any combination of the two measures remains effective, as shown in Table 8. Based on the established seasonal SEIV dynamic model, the prevention and control measures proposed in this study can provide a theoretical reference for the formulation of brucellosis control strategies in Gansu, Guangdong, and Sichuan provinces.

Brucellosis transmission exhibits pronounced seasonal variation trends. However, most existing studies adopt annual data for modeling and prediction, which fails to fully demonstrate the seasonal transmission patterns of brucellosis. Periodic transmission rate have been applied to simulate seasonal prevalent infectious diseases such as schistosomiasis and HFMD [21,45]. In this study, periodic transmission rates were also introduced into the model, and the *MAPE* between the simulated and observed values indicated good fitting performance, thereby verifying the rationality of the seasonal SEIV dynamic model for characterizing the transmission patterns of brucellosis. Additionally, the data on new human brucellosis cases adopted in this study are updated monthly. The control measures derived from the latest data further demonstrate the high practical applicability of the model, laying a theoretical foundation for the formulation of brucellosis prevention and control policies.

The basic reproduction number *R*_0_ characterizes the transmission intensity of an infectious disease. When *R*_0_ > 1, a larger *R*_0_ value corresponds to higher transmissibility and greater difficulty in epidemic control. Li et al. [16] developed a dynamic model to fit annual new human brucellosis cases in 11 provinces of mainland China from 2004 to 2014, estimating the range of *R*_0_ as 1.0227 - 1.8348. Qin et al. [34] established a patch dynamic model to estimate the *R*_0_ of brucellosis transmission in Shanxi and Hebei provinces over the period 2010–2018, and the corresponding *R*_0_ values were estimated at 0.7497 and 0.5022. Gong et al. [46] proposed a sheep-human-environment dynamical model to simulate newly reported human brucellosis cases in Ningxia during 2005–2020, with the *R*_0_ was calculated to be 1.47. Liu et al. [33] established a sheep-to-human transmission model, fitted the number of new human brucellosis cases in Inner Mongolia over 2001–2021, yielding an *R*_0_ value of 2.86. In this study, we adopted periodic transmission rates to calculate that the *R*_0_ for Gansu, Guangdong and Sichuan provinces. The estimated *R*_0_ values are either within or close to the range reported in previous studies for other provincial regions of China.This reveals that our constructed seasonal dynamic model can not only capture periodic epidemic transmission pattern, but also accurately estimate the *R*_0,_ which allows a quantitative evaluation of transmission intensity across different provinces.

This study has several limitations. (1) The seasonal modeling adopted a purely mathematical sinusoidal function as the seasonal forcing term, without incorporating climatic factors into the framework. This specification serves as a minimal-complexity baseline model that mainly captures the first-order characteristics of seasonal fluctuations in brucellosis. Nevertheless, this approach fails to establish mechanistic links with actual climatic variables such as temperature and humidity, which objectively weakens the model’s explanatory power for seasonal driving factors and may constrain the robustness of its long-term predictions. To overcome this limitation, future research will integrate observed climatic data (e.g., temperature and humidity) to develop a mechanism-driven seasonal model, thereby improving both the predictive performance of for brucellosis seasonal variations and the mechanistic interpretability of the underlying drivers. (2) This study neglects the direct infection of cattle, sheep, and humans caused by brucella present in the environment. This simplification, often justified by considerations of parameter identifiability and model parsimony, it inevitably reduces the biological realism of the model. Omission of environmental transmission routes may lead to systematically underestimate of the actual transmission risk of brucellosis. In other words, the quantitative findings presented here should be interpreted as lower-bound estimates of transmission risk, which are useful for assessing minimal resource allocation for interventions but are not suitable for precise predictions of absolute risk. Notably, the current model adopts a modular structure, allowing the environmental transmission compartment to be incorporated without fundamentally restructuring the core transmission framework. (3) This study did not include the effects of animals such as pigs, horses, and dogs on the transmission dynamics of brucellosis. Although these animals are not primary hosts, they may serve as auxiliary or bridge hosts, thereby influencing pathogen maintenance and cross-species transmission. Excluding these factors further reduces the biological realism of the model and may exacerbate the systematic underestimation of transmission risk. Future research will prioritize the identification of auxiliary host species that contribute significantly to infectious disease dynamics and progressively integrate them into the model, with the goal of developing a multi-host transmission dynamics model that encompasses a more complete range of hosts and more comprehensive transmission pathways. Meanwhile, we will continuously optimize the application of mathematical modeling approaches in infectious diseases and propose practical, effective, and implementable control strategies based on the transmission characteristics of brucellosis [47–50].

## Supporting information

S1 Fig Traces for the six parameters associated with the periodic transmission rate of brucellosis and for the initial values of three important compartments in Gansu, Guangdong, and Sichuan provinces. (A) The parameters *a*, *b*, *c*, *a*_*h*_, *b*_*h*_, *c*_*h*_ and the initial values *E*(0), *I*(0), *V*(0) in Gansu province. (B) The parameters *a*, *b*, *c*, *a*_*h*_, *b*_*h*_, *c*_*h*_ and the initial values *E*(0), *I*(0), *V*(0) in Guangdong province. (C) The parameters *a*, *b*, *c*, *a*_*h*_, *b*_*h*_, *c*_*h*_ and the initial values *E*(0), *I*(0), *V*(0) in Sichuan province.
(TIF)

S2 Fig Histograms for the six parameters associated with the periodic transmission rate of brucellosis and for the initial values of three important compartments in Gansu, Guangdong, and Sichuan provinces. (A) The parameters *a*, *b*, *c*, *a*_*h*_, *b*_*h*_, *c*_*h*_ and the initial values *E*(0), *I*(0), *V*(0) in Gansu province. (B) The parameters *a*, *b*, *c*, *a*_*h*_, *b*_*h*_, *c*_*h*_ and the initial values *E*(0), *I*(0), *V*(0) in Guangdong province. (C) The parameters *a*, *b*, *c*, *a*_*h*_, *b*_*h*_, *c*_*h*_ and the initial values *E*(0), *I*(0), *V*(0) in Sichuan province.
(TIF)

S3 Fig Autocorrelation plots for the six parameters associated with the periodic transmission rate of brucellosis and for the initial values of three important compartments in Gansu, Guangdong, and Sichuan provinces. (A) The parameters *a*, *b*, *c*, *a*_*h*_, *b*_*h*_, *c*_*h*_ and the initial values *E*(0), *I*(0), *V*(0) in Gansu province. (B) The parameters *a*, *b*, *c*, *a*_*h*_, *b*_*h*_, *c*_*h*_ and the initial values *E*(0), *I*(0), *V*(0) in Guangdong province. (C) The parameters *a*, *b*, *c*, *a*_*h*_, *b*_*h*_, *c*_*h*_ and the initial values *E*(0), *I*(0), *V*(0) in Sichuan province.
(TIF)

S4 Fig Posterior correlation matrix of the six parameters associated with the periodic transmission rate of brucellosis and the initial values of three important compartments in Gansu, Guangdong, and Sichuan provinces. (A) The parameters *a*, *b*, *c*, *a*_*h*_, *b*_*h*_, *c*_*h*_ and the initial values *E*(0), *I*(0), *V*(0) in Gansu province. (B) The parameters *a*, *b*, *c*, *a*_*h*_, *b*_*h*_, *c*_*h*_ and the initial values *E*(0), *I*(0), *V*(0) in Guangdong province. (C) The parameters *a*, *b*, *c*, *a*_*h*_, *b*_*h*_, *c*_*h*_ and the initial values *E*(0), *I*(0), *V*(0) in Sichuan province.
(TIF)

S5 Fig The model separately fitted the trends of new human brucellosis cases in Gansu, Guangdong, and Sichuan provinces from 2021 to 2023, and predicted the trends for the year 2024. (A) The new human brucellosis cases in Gansu province. (B) The new human brucellosis cases in Guangdong province. (C) The new human brucellosis cases in Sichuan province. Reddish-brown solid dots indicate actual values, while orange, blue, and green curves represent the model-fitted values for Gansu, Guangdong and Sichuan provinces, respectively. The light-colored areas around the curves denote the 95% confidence intervals of the fitted curves.
(TIF)

S6 Fig ACF and PACF plots for new human brucellosis cases in Gansu, Guangdong, and Sichuan provinces from 2021 to 2024. (A) ACF and PACF plots for new human brucellosis cases in Gansu province. (B) ACF and PACF plots for new human brucellosis cases in Guangdong province. (C) ACF and PACF plots for new human brucellosis cases in Sichuan province.
(TIF)

S7 Fig The optimal time-series model simulates new human brucellosis cases from 2021 to 2024 and predicts trends for 2025–2029. (A) Trends in new human brucellosis cases in Gansu province. (B) Trends in new human brucellosis cases in Guangdong province. (C) Trends in new human brucellosis cases in Sichuan province. The solid orange, blue, and green lines represent the model-simulated values for Gansu, Guangdong, and Sichuan provinces, respectively, while the dashed lines represent the 95% confidence intervals of the simulated values. The dark blue area indicates the 50% confidence interval of the predicted curve, and the gray area represents the 95% confidence interval of the predicted values.
(TIF)

S8 Fig Residual plot of actual values versus SARIMA model-predicted values for Gansu, Guangdong, and Sichuan provinces. (TIF)

S9 Fig The effects of parameters variations on the number of infected sheep/cattle in Gansu, Guangdong, and Sichuan provinces. (A), (B), (C) and (D) The effects of *f*, *v*, *δ*, and *A* on the infected sheep/cattle in Gansu province, respectively. (E), (F), (G) and (H) The effects of *f*, *v*, *δ*, and *A* on the infected sheep/cattle in Guangdong province, respectively. (I), (J), (K) and (L) The effects of *f*, *v*, *δ*, and *A* on the infected sheep/cattle in Sichuan province, respectively.
(TIF)

S10 Fig The effects of parameters variations on the number of exposed sheep/cattle in Gansu, Guangdong, and Sichuan provinces. (A), (B), (C) and (D) The effects of *f*, *v*, *δ*, and *A* on the exposed sheep/cattle in Gansu province, respectively. (E), (F), (G) and (H) The effects of *f*, *v*, *δ*, and *A* on the exposed sheep/cattle in Guangdong province, respectively. (I), (J), (K) and (L) The effects of *f*, *v*, *δ*, and *A* on the exposed sheep/cattle in Sichuan province, respectively.
(TIF)

S11 Fig The effects of parameters variations on the number of vaccinated sheep/cattle in Gansu, Guangdong, and Sichuan provinces. (A), (B), (C) and (D) The effects of *f*, *v*, *δ*, and *A* on the vaccinated sheep/cattle in Gansu province, respectively. (E), (F), (G) and (H) The effects of *f*, *v*, *δ*, and *A* on the vaccinated sheep/cattle in Guangdong province, respectively. (I), (J), (K) and (L) The effects of *f*, *v*, *δ*, and *A* on the vaccinated sheep/cattle in Sichuan province, respectively.
(TIF)

S1 File SARIMA model construction procedure. S6 Fig. ACF and PACF plots for new human brucellosis cases in Gansu, Guangdong, and Sichuan provinces from 2021 to 2024. (A) ACF and PACF plots for new human brucellosis cases in Gansu province. (B) ACF and PACF plots for new human brucellosis cases in Guangdong province. (C) ACF and PACF plots for new human brucellosis cases in Sichuan province. Table A. Model results. S7 Fig. The optimal time-series model simulates new human brucellosis cases from 2021 to 2024 and predicts trends for 2025–2029. (A) Trends in new human brucellosis cases in Gansu province. (B) Trends in new human brucellosis cases in Guangdong province. (C) Trends in new human brucellosis cases in Sichuan province. The solid orange, blue, and green lines represent the model-simulated values for Gansu, Guangdong, and Sichuan provinces, respectively, while the dashed lines represent the 95% confidence intervals of the simulated values. The dark blue area indicates the 50% confidence interval of the predicted curve, and the gray area represents the 95% confidence interval of the predicted values. S8 Fig. Residual plot of actual values versus SARIMA model-predicted values for Gansu, Guangdong, and Sichuan provinces.
(DOCX)

S1 TableAcceptance rates, ESS, and \stackrel∧R values for multiple chains.(DOCX)

S2 Table Table A. The values of each parameter and the initial values of each compartment were determined by fitting the model to the new human brucellosis cases in Gansu province from 2021 to 2024. **Table B.** The values of each parameter and the initial values of each compartment were determined by fitting the model to the new human brucellosis cases in Guangdong province from 2021 to 2024. **Table C.** The values of each parameter and the initial values of each compartment were determined by fitting the model to the new human brucellosis cases in Sichuan province from 2021 to 2024.
(DOCX)
