# Supplementary material for: Seasonal dynamic modeling for real-time prediction of human brucellosis epidemiological trends in Gansu, Guangdong and Sichuan Provinces, China
Source: PLoS Negl Trop Dis. 2026 Jun 25;20(6):e0014443. doi: 10.1371/journal.pntd.0014443 (PMC13298781; doi:10.1371/journal.pntd.0014443)
Supplement: S1 File — S6 Fig. ACF and PACF plots for new human brucellosis cases in Gansu, Guangdong, and Sichuan provinces from 2021 to 2024. (A) ACF and PACF plots for new human brucellosis cases in Gansu province. (B) ACF and PACF plots for new human brucellosis cases in Guangdong province. (C) ACF and PACF plots for new human brucellosis cases in Sichuan province. Table A. Model results. S7 Fig. The optimal time-series model simulates new human brucellosis cases from 2021 to 2024 and predicts trends for 2025–2029. (A) Trends in new human brucellosis cases in Gansu province. (B) Trends in new human brucellosis cases in Guangdong province. (C) Trends in new human brucellosis cases in Sichuan province. The solid orange, blue, and green lines represent the model-simulated values for Gansu, Guangdong, and Sichuan provinces, respectively, while the dashed lines represent the 95% confidence intervals of the simulated values. The dark blue area indicates the 50% confidence interval of the predicted curve, and the gray area represents the 95% confidence interval of the predicted values. S8 Fig. Residual plot of actual values versus SARIMA model-predicted values for Gansu, Guangdong, and Sichuan provinces. (DOCX) [file pntd.0014443.s012.docx]

**Supporting information**

**S1 Text. SARIMA model construction procedure.**

**1. Introduction to the SARIMA Model**

The Seasonal Autoregressive Integrated Moving Average (SARIMA) model is capable of fully capturing the inherent information of a time series that exhibits complex correlations among seasonality, trends, and random errors. Newly reported human brucellosis cases in Gansu, Guangdong, and Sichuan provinces show a rising trend and obvious seasonal periodicity. Given the multiplicative relationship between short-term autocorrelation and seasonal effects, the model is formulated as SARIMA(*p*, *d*, *q*)×(*P*, *D*, *Q*)*s*, with its specific structure presented as follows:

among them

Let$X_{t}$denote a non-stationary time series at time *t*, let$\varepsilon_{t}$denote a zero-mean white noise sequence, and let *s* denote the seasonal period. When time series under study exhibits seasonal patterns and the data recorded on a monthly basis, we set *s*=12. Let *B* denote the lag operator. In the general model, *p* denotes the autoregressive order, *d* denotes the differencing order, and *q* denotes the moving average order,$\theta(B)$denotes the *q*-th order moving average coefficient polynomial, and denotes the *p*-th order autoregressive coefficient polynomial. In the seasonal model, *P* represents the seasonal autoregressive order, *D* represents the seasonal differencing order, and *Q* represents the seasonal moving average order, $\theta_{S}(B)$denotes the *Q*-th order seasonal moving average coefficient polynomial, anddenotes the *P*-th order seasonal autoregressive coefficient polynomial.

# 2. Model Application

(1) Use the Augmented Dickey-Fuller (ADF) test and the Kwiatkowski-Phillips-Schmidt-Shin (KPSS) test to examine the stationary of the data ; if not, differencing should be performed to render it stationary. (2) Plot the autocorrelation function (ACF) and partial autocorrelation function (PACF) graphs of the stationary time series to select the optimal orders for the SARIMA model. Determine the values of *p*, *d*, *q* (non-seasonal part) and *P*, *D*, *Q* (seasonal part), thereby constructing the SARIMA(*p*, *d*, *q*)×(*P*, *D*, *Q*)*s* model. (3) The Akaike Information Criterion (AIC) and Bayesian Information Criterion (BIC) were adopted to select the optimal SARIMA model, which balances good fitting performance and the avoidance of overfitting. (4) The data were fitted with optimal SARIMA model, and the model performance was verified by residual analysis and the Ljung-Box test. (5) The validated SARIMA model was adopted to predict the number of new human brucellosis cases, and the prediction results were visualized to intuitively reflect the evolutionary trend of the time series.

**3. Results**

(1) The ADF test was performed on the time series of new human brucellosis cases in Gansu, Guangdong, and Sichuan provinces. All ADF tests returned P < 0.05, whereas all KPSS tests yielded *P* > 0.05. These results confirm that the time series are stationary, indicating that no non-seasonal differencing is required. (2) According to the ACF and PACF plots (S6 Fig), and the principle of minimum AIC and BIC values, the optimal model were identified as follows: SARIMA(0, 0, 1)(1, 1, 0)₁₂ for Gansu Province, SARIMA(1, 0, 0)(0, 1, 0)₁₂ for Guangdong Province, and SARIMA(0, 0, 0)(0, 1, 1)₁₂ for Sichuan Province. (3) A Ljung–Box test was conducted to examine the whiteness of model residuals. All results showed P > 0.05, indicating that the residuals followed a white noise process and that the temporal information was sufficiently extracted. Detailed results are summarized in Table A. (4) Using the established optimal SARIMA models, the simulated number of new human brucellosis cases in Gansu, Guangdong, and Sichuan provinces from 2021 to 2024 all exhibited an upward trend, with corresponding MAPE values of 15.12%, 20.45%, and 23.84%, as illustrated in S7 Fig. The residuals between the observed and model-fitted values are displayed in S8 Fig. Meanwhile, short-term forecastting suggests that new brucellosis cases will maintain a relatively stable trend over the next five years.

**Table A.** Model results.

| **Item** | **Gansu** | **Guangdong** | **Sichuan** |
| --- | --- | --- | --- |
| ADF test | *P* = 0.0218 | *P* = 0.0430 | *P* = 0.0417 |
| KPSS test | *P* > 0.05 | *P* > 0.05 | *P* > 0.05 |
| Optimal model | SARIMA(0, 0, 1)(1, 1, 0)_12_ | SARIMA(1, 0, 0)(0, 1, 0)_12_ | SARIMA(0, 0, 0)(0, 1, 1)_12_ |
| AIC | 439.97 | 305.92 | 270.90 |
| BIC | 444.72 | 309.08 | 275.66 |
| Residual analysis | *P* = 0.1716 | *P* = 0.1659 | *P* = 0.0514 |
