# Supplementary material for: Seasonal dynamic modeling for real-time prediction of human brucellosis epidemiological trends in Gansu, Guangdong and Sichuan Provinces, China
Source: PLoS Negl Trop Dis. 2026 Jun 25;20(6):e0014443. doi: 10.1371/journal.pntd.0014443 (PMC13298781; doi:10.1371/journal.pntd.0014443)
Supplement: S1 Table — (DOCX) [file pntd.0014443.s013.docx]

**S1 Table. Acceptance rates, ESS, and** $\mathop{R}\limits^{\wedge}$ **values for multiple chains.**

| **Items** | | **Gansu** | | | $\mathop{R}\limits^{\wedge}$ |  | **Guangdong** | | | $\mathop{R}\limits^{\wedge}$ |  | **Sichuan** | | | $\mathop{R}\limits^{\wedge}$ |
| --- | --- | --- | --- | --- | --- | --- | --- | --- | --- | --- | --- | --- | --- | --- | --- |
|  |  | **Trace 1** | **Trace 2** | **Trace 3** |  |  | **Trace 1** | **Trace 2** | **Trace 3** |  |  | **Trace 1** | **Trace 2** | **Trace 3** |  |
| Acceptance rate | | 68.53% | 68.41% | 68.88% | - |  | 66.55% | 66.98% | 65.73% | - |  | 58.15% | 54.60% | 52.98% | - |
| ESS | *a* | 1,118.98 | 882.46 | 1,110.26 | 0.0020 |  | 790.25 | 817.12 | 733.53 | 0.0974 |  | 500.41 | 345.39 | 425.54 | 0.0108 |
|  | *b* | 740.77 | 800.17 | 738.88 | 1.0005 |  | 362.08 | 566.84 | 438.07 | 1.0025 |  | 295.58 | 125.81 | 219.05 | 1.0016 |
|  | *c* | 1,001.82 | 739.25 | 994.65 | 1.0001 |  | 432.28 | 444.94 | 327.17 | 1.0009 |  | 185.37 | 175.82 | 184.78 | 1.0023 |
|  | *a_h_* | 964.75 | 763.05 | 1,089.21 | 0.0008 |  | 593.64 | 702.78 | 737.91 | 0.0018 |  | 371.49 | 198.24 | 355.12 | 0.0002 |
|  | *b_h_* | 714.72 | 742.35 | 654.83 | 1.0003 |  | 551.22 | 652.98 | 461.69 | 1.0001 |  | 208.18 | 258.61 | 213.59 | 1.0015 |
|  | *c_h_* | 952.42 | 836.60 | 1,132.45 | 1.0004 |  | 327.28 | 580.93 | 413.25 | 1.0022 |  | 301.26 | 122.97 | 191.11 | 1.0009 |
|  | *E*(0) | 935.01 | 909.37 | 1,054.66 | 1.0006 |  | 784.90 | 820.65 | 676.60 | 1.0030 |  | 501.05 | 505.64 | 334.50 | 1.0000 |
|  | *I*(0) | 1,056.91 | 750.66 | 999.70 | 1.0011 |  | 676.31 | 901.06 | 713.96 | 1.0017 |  | 653.21 | 519.23 | 435.16 | 1.0074 |
|  | *V*(0) | 960.08 | 978.06 | 921.71 | 1.0008 |  | 824.64 | 859.12 | 765.70 | 1.0011 |  | 435.94 | 448.30 | 368.73 | 1.0002 |
