# Supplementary material for: Seasonal dynamic modeling for real-time prediction of human brucellosis epidemiological trends in Gansu, Guangdong and Sichuan Provinces, China
Source: PLoS Negl Trop Dis. 2026 Jun 25;20(6):e0014443. doi: 10.1371/journal.pntd.0014443 (PMC13298781; doi:10.1371/journal.pntd.0014443)
Supplement: S2 Table — The values of each parameter and the initial values of each compartment were determined by fitting the model to the new human brucellosis cases in Gansu province from 2021 to 2024. Table B. The values of each parameter and the initial values of each compartment were determined by fitting the model to the new human brucellosis cases in Guangdong province from 2021 to 2024. Table C. The values of each parameter and the initial values of each compartment were determined by fitting the model to the new human brucellosis cases in Sichuan province from 2021 to 2024. (DOCX) [file pntd.0014443.s014.docx]

**S2 Table.**

**Table A.** The values of each parameter and the initial values of each compartment were determined by fitting the model to the new human brucellosis cases in Gansu province from 2021 to 2024.

| **Dataset span** | | | |  |  | ****Parameter values**** | |  | ****Initial compartment values**** | |
| --- | --- | --- | --- | --- | --- | --- | --- | --- | --- | --- |
| **Month** | **2021** | **2022** | **2023** | **2024** |  | **Parameter** | **Value** |  | **Compartment** | **Initial value** |
| 1 | 283 | 421 | 221 | 390 |  | *A* | 808,426 |  | *S*(0) | 26,642,889 |
| 2 | 232 | 333 | 476 | 325 |  | *$\mu$* | 0.0185 |  | *V*(0) | 44,992  (95%CI: 42,084 - 47,900) |
| 3 | 384 | 417 | 488 | 499 |  | *ƒ* | 0.0163 |  | *E*(0) | 15,038  (95%CI: 12,130 - 17,946) |
| 4 | 447 | 502 | 506 | 543 |  | *δ* | 0.0333 |  | *I*(0) | 35,081  (95%CI: 32,211 - 37,951) |
| 5 | 460 | 697 | 632 | 569 |  | *ν* | 0.0236 |  | *S_h_*(0) | 11,952,499 |
| 6 | 531 | 696 | 661 | 609 |  | *ε* | 0.0150 |  | *S_ha_*(0) | 283 |
| 7 | 560 | 641 | 663 | 765 |  | *λ* | 2 |  | *S_hc_*(0) | 44 |
| 8 | 458 | 620 | 772 | 644 |  | *a* | 1.6920×10^-9^  (95%CI: 1.6624×10^-9^ - 1.7215×10^-9^) |  |  |  |
| 9 | 341 | 466 | 540 | 452 |  | *b* | 10.1990  (95%CI: 9.4590 - 10.9390) |  |  |  |
| 10 | 211 | 221 | 426 | 314 |  | *c* | 0.6767  (95%CI: 0.6094 - 0.7440) |  |  |  |
| 11 | 263 | 222 | 365 | 417 |  | *B* | 17770 |  |  |  |
| 12 | 392 | 142 | 386 | 396 |  | *$\mu_{1}$* | 0.00069 |  |  |  |
|  |  |  |  |  |  | *η* | 0.0500 |  |  |  |
|  |  |  |  |  |  | *$\omega_{h}$* | 0.1667 |  |  |  |
|  |  |  |  |  |  | *a_h_* | 1.8350×10^-10^  (95%CI: 1.7149×10^-10^ - 1.9551×10^-10^) |  |  |  |
|  |  |  |  |  |  | *b_h_* | 0.46715  (95%CI: 0.4199 - 0.5143) |  |  |  |
|  |  |  |  |  |  | *c_h_* | 2.5752  (95%CI: 2.4648 - 2.6856) |  |  |  |

**Table B.** The values of each parameter and the initial values of each compartment were determined by fitting the model to the new human brucellosis cases in Guangdong province from 2021 to 2024.

| **Dataset span** | | | |  |  | ****Parameter values**** | |  | ****Initial compartment values**** | |
| --- | --- | --- | --- | --- | --- | --- | --- | --- | --- | --- |
| **Month** | **2021** | **2022** | **2023** | **2024** |  | **Parameter** | **Value** |  | **Compartment** | **Initial value** |
| 1 | 30 | 31 | 10 | 32 |  | *A* | 28,142 |  | *S*(0) | 2,158,972 |
| 2 | 27 | 21 | 44 | 52 |  | *$\mu$* | 0.0185 |  | *V*(0) | 3,521  (95%CI: 3,233 - 3,809) |
| 3 | 63 | 44 | 68 | 68 |  | *ƒ* | 0.0163 |  | *E*(0) | 1,497  (95%CI: 1,209 - 1,785) |
| 4 | 78 | 50 | 65 | 113 |  | *δ* | 0.0333 |  | *I*(0) | 3,006  (95%CI: 2,430 - 3,582) |
| 5 | 76 | 77 | 77 | 115 |  | *ν* | 0.0236 |  | *S_h_*(0) | 32,576,436 |
| 6 | 47 | 66 | 73 | 136 |  | *ε* | 0.0150 |  | *S_ha_*(0) | 30 |
| 7 | 36 | 58 | 90 | 123 |  | *λ* | 2 |  | *S_hc_*(0) | 5 |
| 8 | 33 | 47 | 79 | 92 |  | *a* | 3.6357×10^-8^  (95%CI: 3.6292×10^-8^ - 3.6422×10^-8^ ) |  |  |  |
| 9 | 21 | 43 | 51 | 59 |  | *b* | 3.9255  (95%CI: 2.8731 - 4.9779) |  |  |  |
| 10 | 14 | 31 | 33 | 45 |  | *c* | 1.7464  (95%CI: 1.5398 - 1.9530) |  |  |  |
| 11 | 16 | 27 | 30 | 29 |  | *B* | 90,495 |  |  |  |
| 12 | 14 | 11 | 30 | 32 |  | *$\mu_{1}$* | 0.00046 |  |  |  |
|  |  |  |  |  |  | *η* | 0.0500 |  |  |  |
|  |  |  |  |  |  | *$\omega_{h}$* | 0.1667 |  |  |  |
|  |  |  |  |  |  | *a_h_* | 2.5005×10^-10^  (95%CI: 2.2301×10^-10^ - 2.7709×10^-10^) |  |  |  |
|  |  |  |  |  |  | *b_h_* | 0.6249  (95%CI: 0.5656 - 0.6842) |  |  |  |
|  |  |  |  |  |  | *c_h_* | 10.9500  (95%CI: 10.7764 - 11.1236) |  |  |  |

**Table C.** The values of each parameter and the initial values of each compartment were determined by fitting the model to the new human brucellosis cases in Sichuan province from 2021 to 2024.

| **Dataset span** | | | |  |  | ****Parameter values**** | |  | ****Initial compartment values**** | |
| --- | --- | --- | --- | --- | --- | --- | --- | --- | --- | --- |
| **Month** | **2021** | **2022** | **2023** | **2024** |  | **Parameter** | **Value** |  | **Compartment** | **Initial value** |
| 1 | 12 | 15 | 15 | 19 |  | *A* | 406,571 |  | *S*(0) | 23,965,208 |
| 2 | 21 | 11 | 26 | 37 |  | *$\mu$* | 0.0185 |  | *V*(0) | 40,845  (95%CI: 35,276 - 46,414) |
| 3 | 16 | 32 | 42 | 38 |  | *ƒ* | 0.0163 |  | *E*(0) | 14,884  (95%CI: 12,073 - 17,695) |
| 4 | 30 | 23 | 43 | 64 |  | *δ* | 0.0333 |  | *I*(0) | 30,063  (95%CI: 24,362 - 35,764) |
| 5 | 32 | 29 | 70 | 62 |  | *ν* | 0.0236 |  | *S_h_*(0) | 36,208,956 |
| 6 | 25 | 39 | 44 | 60 |  | *ε* | 0.0150 |  | *S_ha_*(0) | 12 |
| 7 | 14 | 30 | 53 | 65 |  | *λ* | 2 |  | *S_hc_*(0) | 2 |
| 8 | 14 | 33 | 44 | 51 |  | *a* | 3.4040×10^-9^  (95%CI: 3.2441×10^-9^ - 3.5639×10^-9^) |  |  |  |
| 9 | 8 | 26 | 30 | 35 |  | *b* | 2.8113  (95%CI: 1.4356 - 4.1870) |  |  |  |
| 10 | 14 | 16 | 31 | 28 |  | *c* | 8.1169  (95%CI: 7.6462 - 8.5876) |  |  |  |
| 11 | 7 | 10 | 26 | 22 |  | *B* | 48,972 |  |  |  |
| 12 | 6 | 15 | 24 | 30 |  | *$\mu_{1}$* | 0.00071 |  |  |  |
|  |  |  |  |  |  | *η* | 0.0500 |  |  |  |
|  |  |  |  |  |  | *$\omega_{h}$* | 0.1667 |  |  |  |
|  |  |  |  |  |  | *a_h_* | 1.1895×10^-11^  (95%CI: 1.1769×10^-11^ - 1.2021×10^-11^ ) |  |  |  |
|  |  |  |  |  |  | *b_h_* | 0.5017  (95%CI: 0.3991 - 0.6043) |  |  |  |
|  |  |  |  |  |  | *c_h_* | 4.7185  (95%CI: 4.3757 - 5.0613) |  |  |  |
